# Supplementary figures and images for: m6A modifications regulate intestinal immunity and rotavirus infection
Source: eLife. 2022 Jan 31;11:e73628. doi: 10.7554/eLife.73628 (PMC8860440; doi:10.7554/eLife.73628)

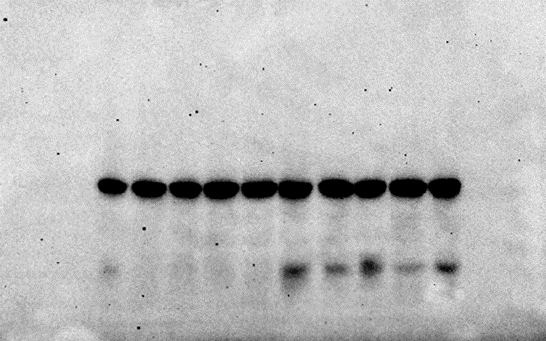

Supplement: Source data 1. [file elife-73628-data1.zip › Figure/Source data to Figure4/figure 4a-FTO-raw.png]

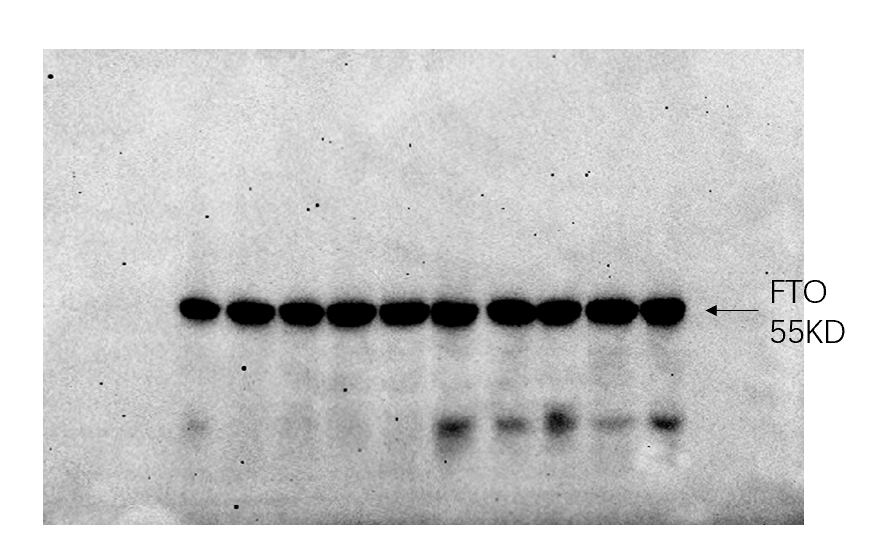

Supplement: Source data 1. [file elife-73628-data1.zip › Figure/Source data to Figure4/figure 4a-FTO.png]

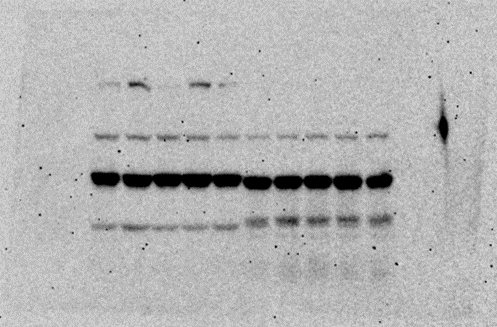

Supplement: Source data 1. [file elife-73628-data1.zip › Figure/Source data to Figure4/figure 4a-METTL14-raw.png]

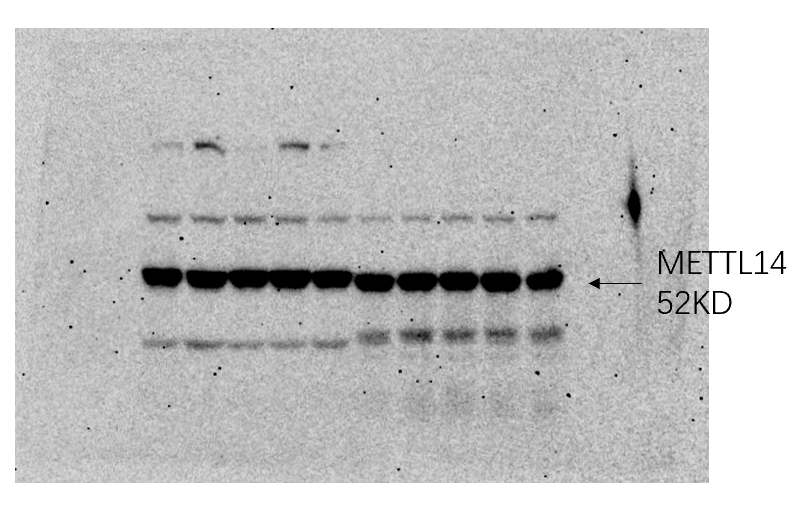

Supplement: Source data 1. [file elife-73628-data1.zip › Figure/Source data to Figure4/figure 4a-METTL14.png]

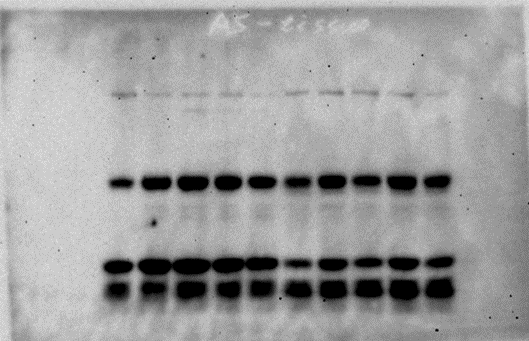

Supplement: Source data 1. [file elife-73628-data1.zip › Figure/Source data to Figure4/figure 4a-METTL3-raw.png]

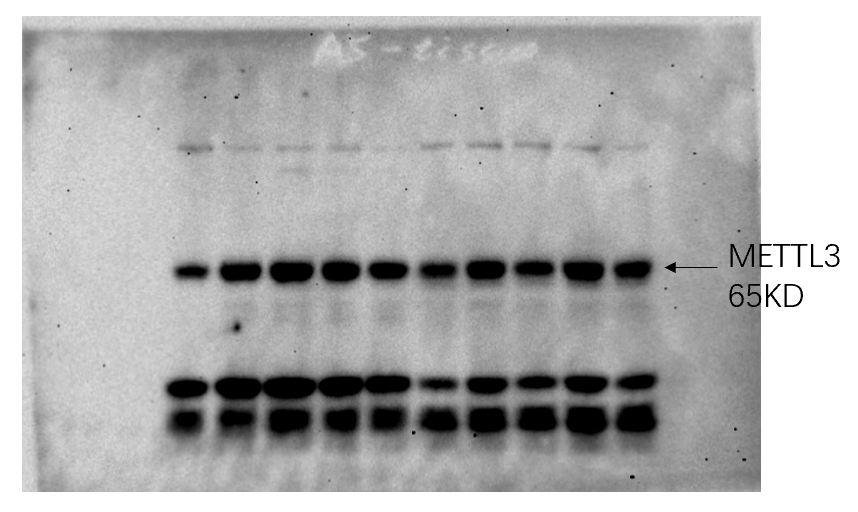

Supplement: Source data 1. [file elife-73628-data1.zip › Figure/Source data to Figure4/figure 4a-METTL3.png]

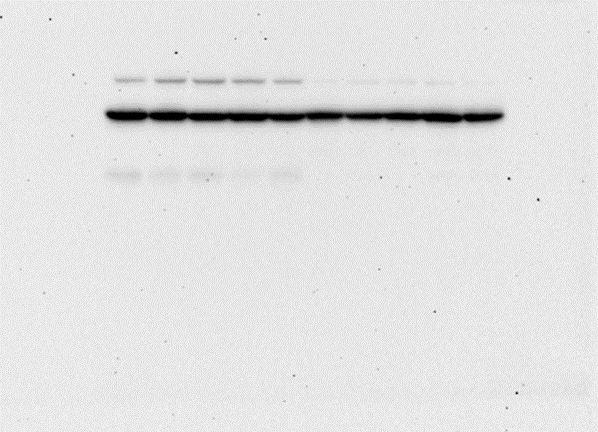

Supplement: Source data 1. [file elife-73628-data1.zip › Figure/Source data to Figure4/figure 4a-actin-raw.png]

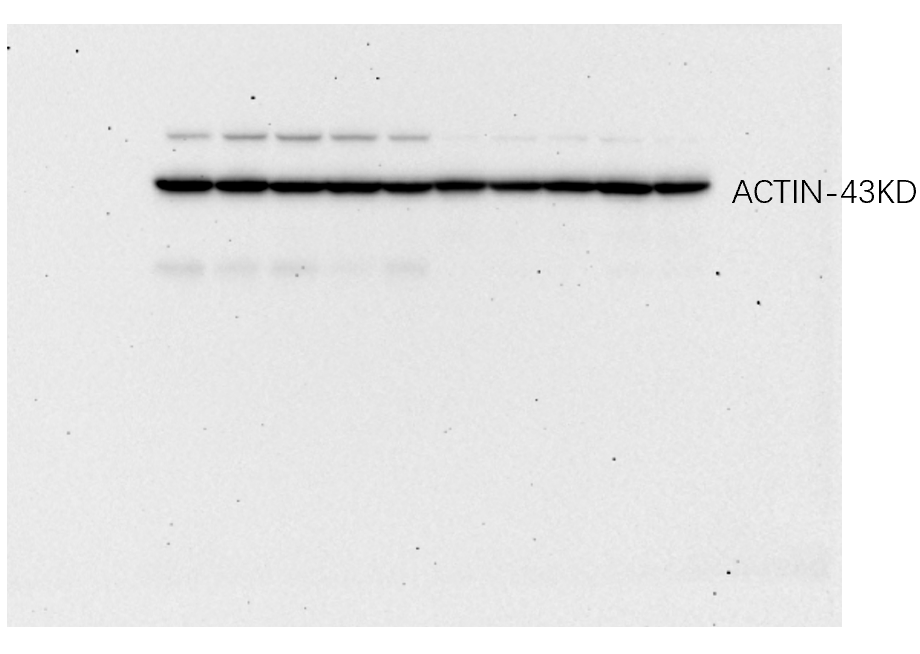

Supplement: Source data 1. [file elife-73628-data1.zip › Figure/Source data to Figure4/figure 4a-actin.png]

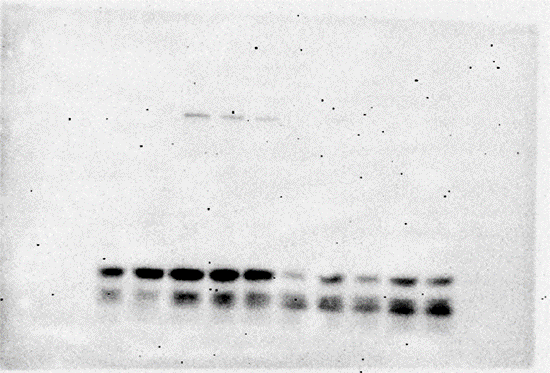

Supplement: Source data 1. [file elife-73628-data1.zip › Figure/Source data to Figure4/figure 4a-alkbh5-raw.png]

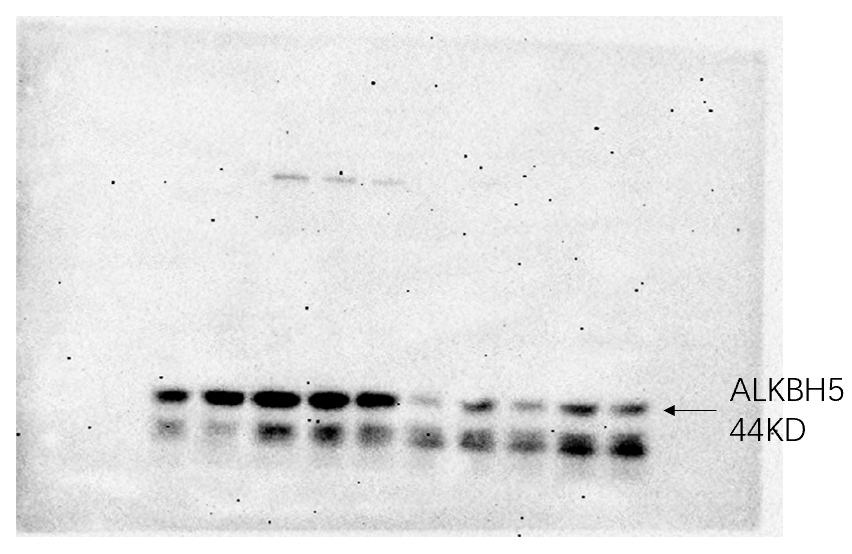

Supplement: Source data 1. [file elife-73628-data1.zip › Figure/Source data to Figure4/figure 4a-alkbh5.png]

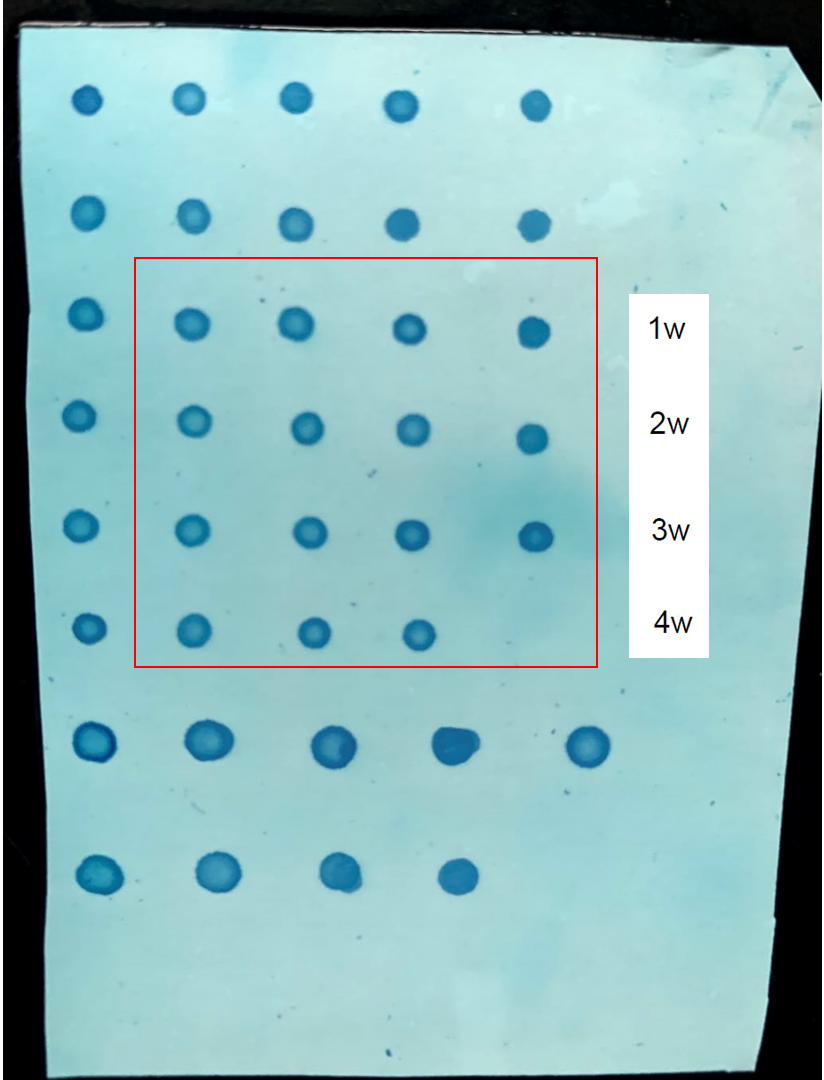

Supplement: Source data 1. [file elife-73628-data1.zip › Figure/Source data to Figure1/figure 1a-methyl blue.png]

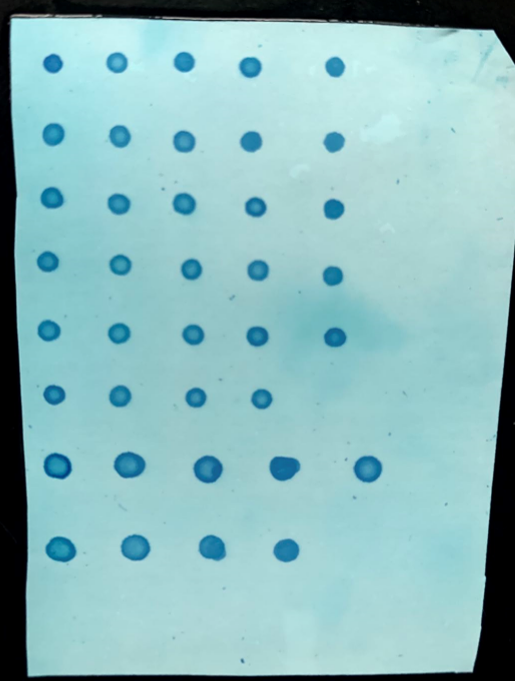

Supplement: Source data 1. [file elife-73628-data1.zip › Figure/Source data to Figure1/figure 1a-raw-methyl blue.png]

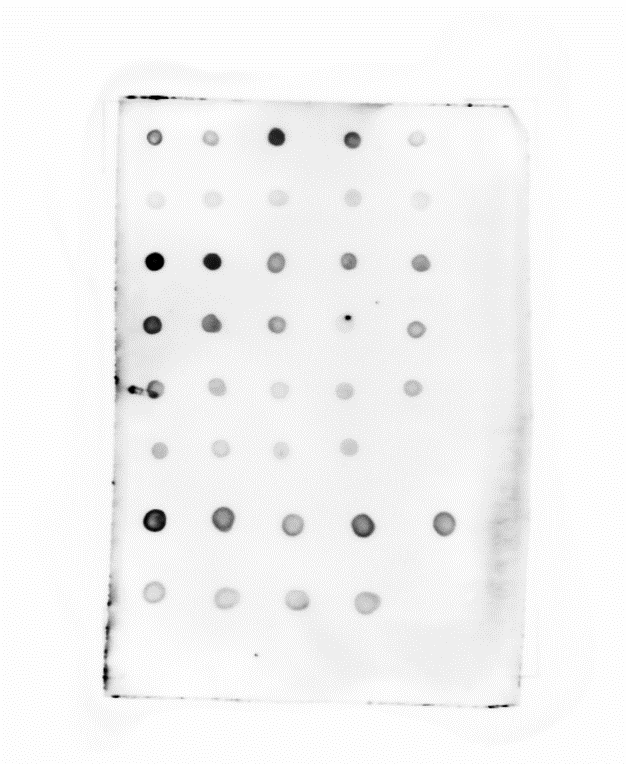

Supplement: Source data 1. [file elife-73628-data1.zip › Figure/Source data to Figure1/figure 1a-raw.png]

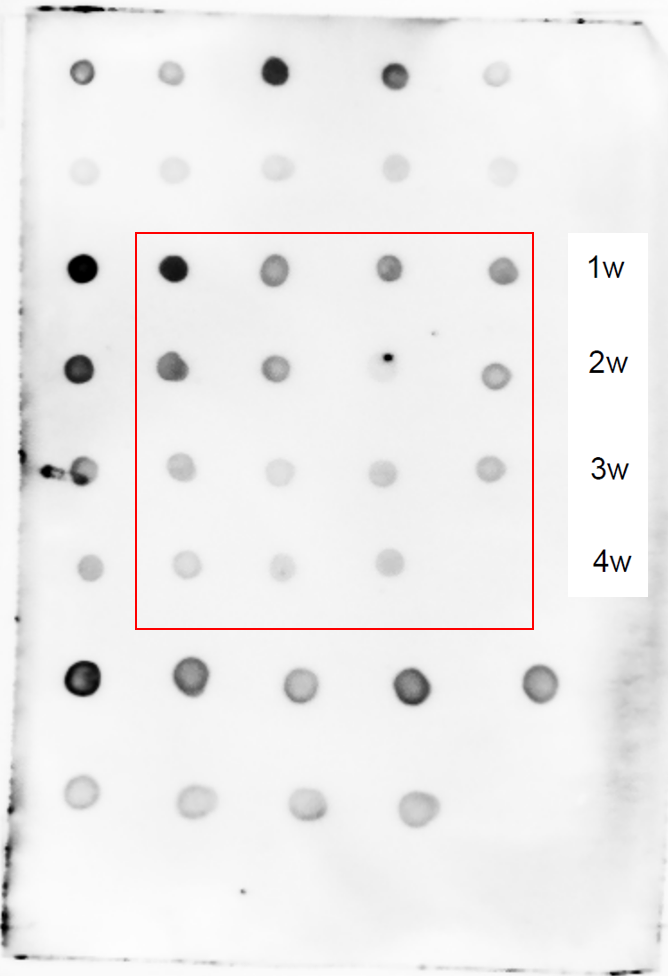

Supplement: Source data 1. [file elife-73628-data1.zip › Figure/Source data to Figure1/figure 1a.png]

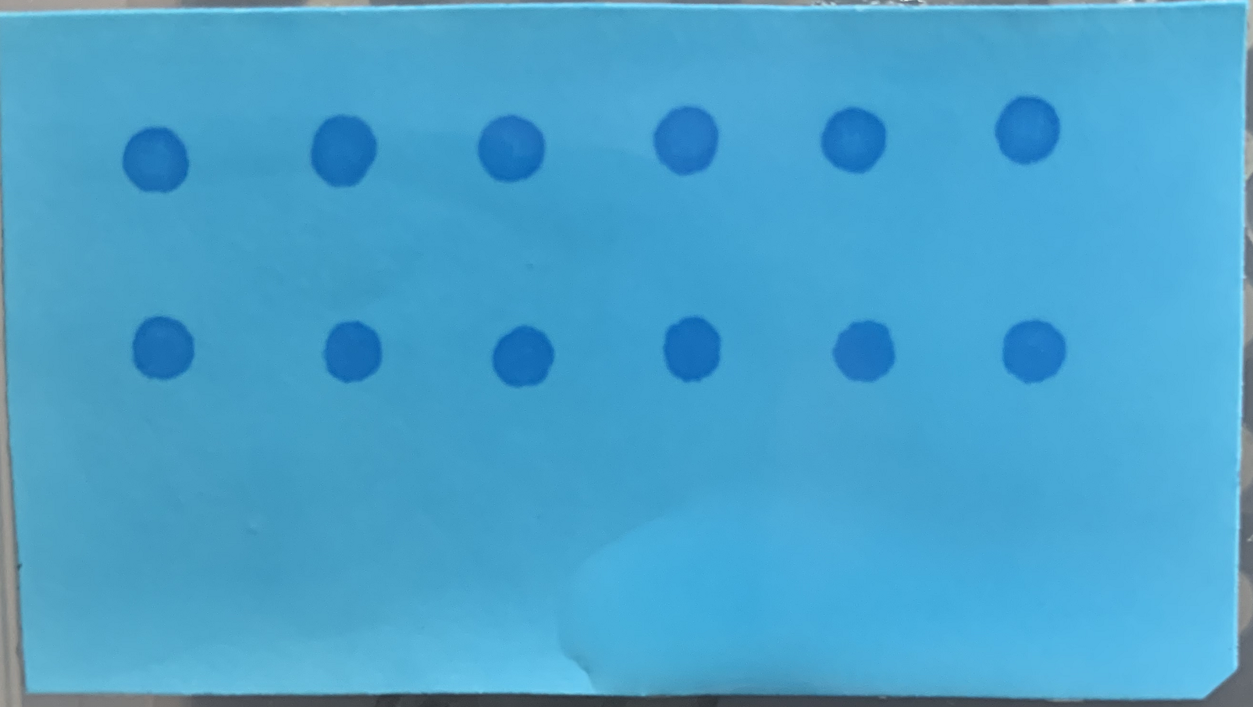

Supplement: Source data 1. [file elife-73628-data1.zip › Figure/Source data to Figure1/figure 1e-methyl blue-raw.png]

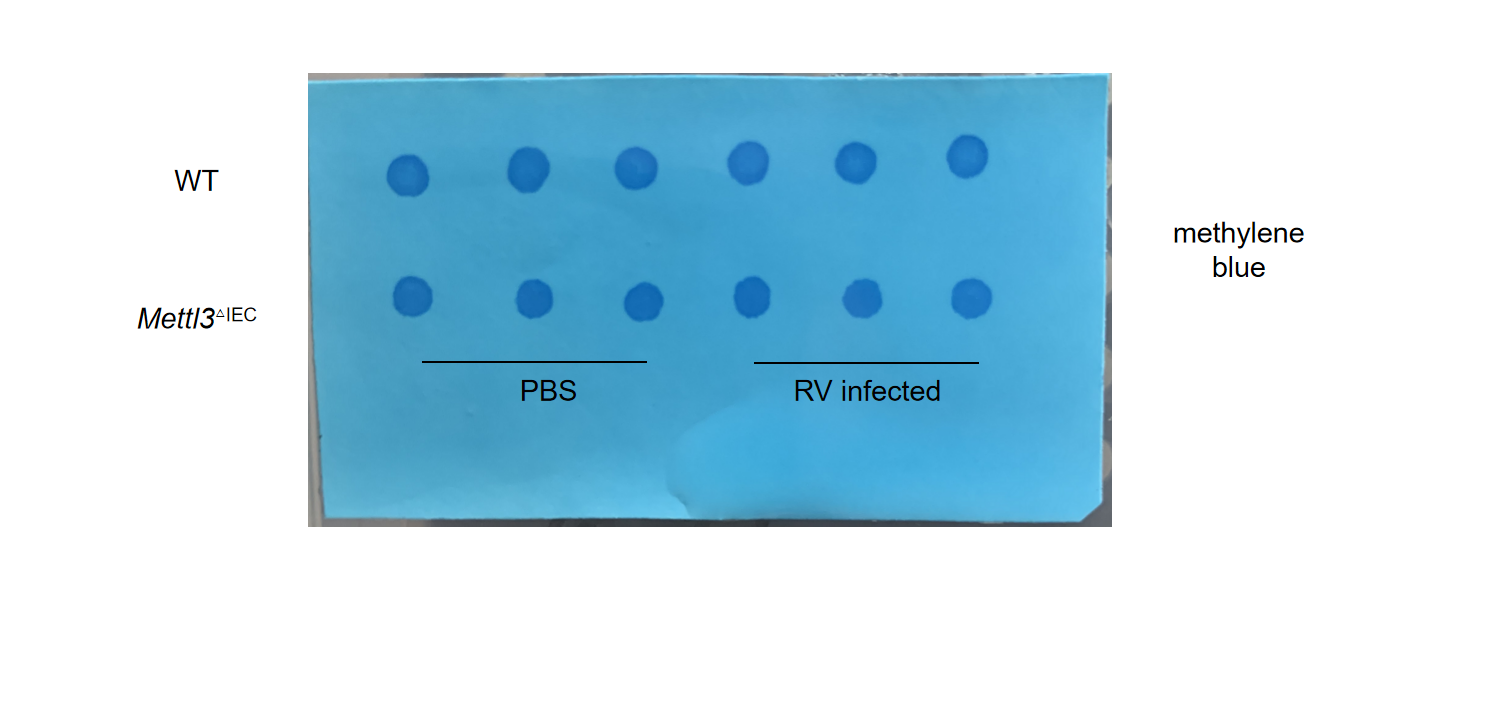

Supplement: Source data 1. [file elife-73628-data1.zip › Figure/Source data to Figure1/figure 1e-methyl blue.png]

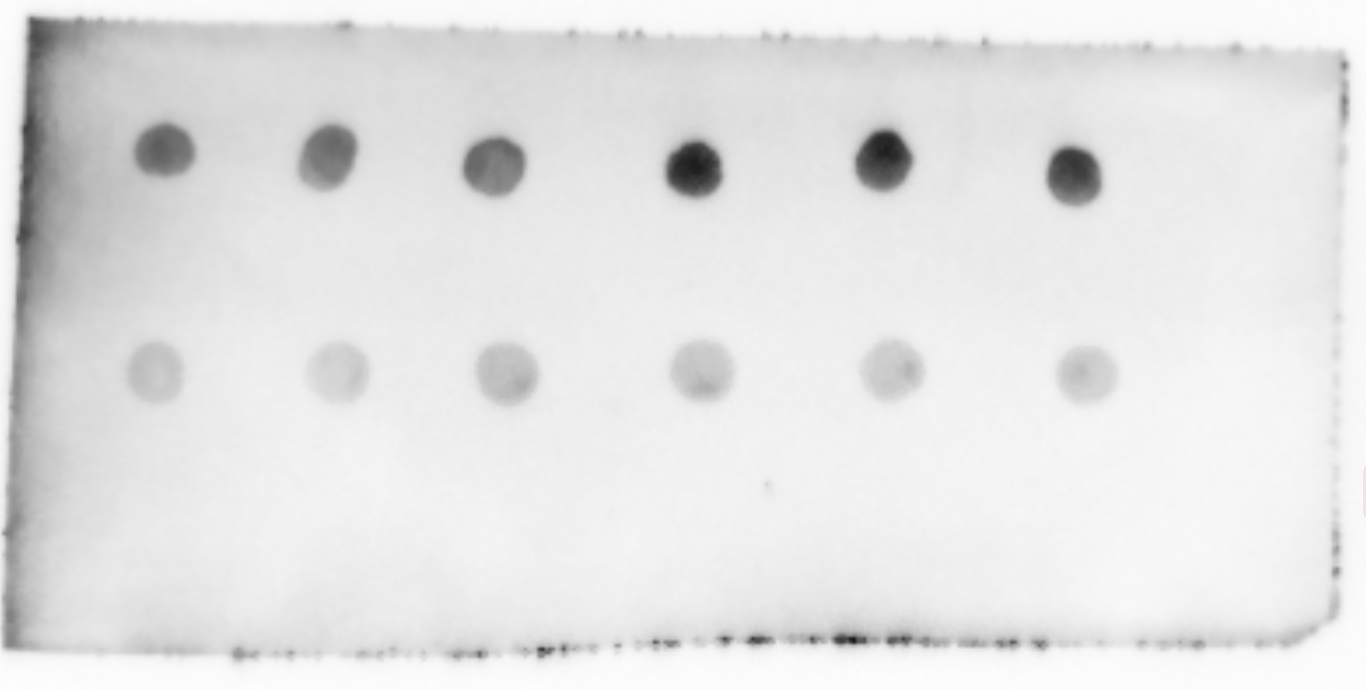

Supplement: Source data 1. [file elife-73628-data1.zip › Figure/Source data to Figure1/figure 1e-raw.png]

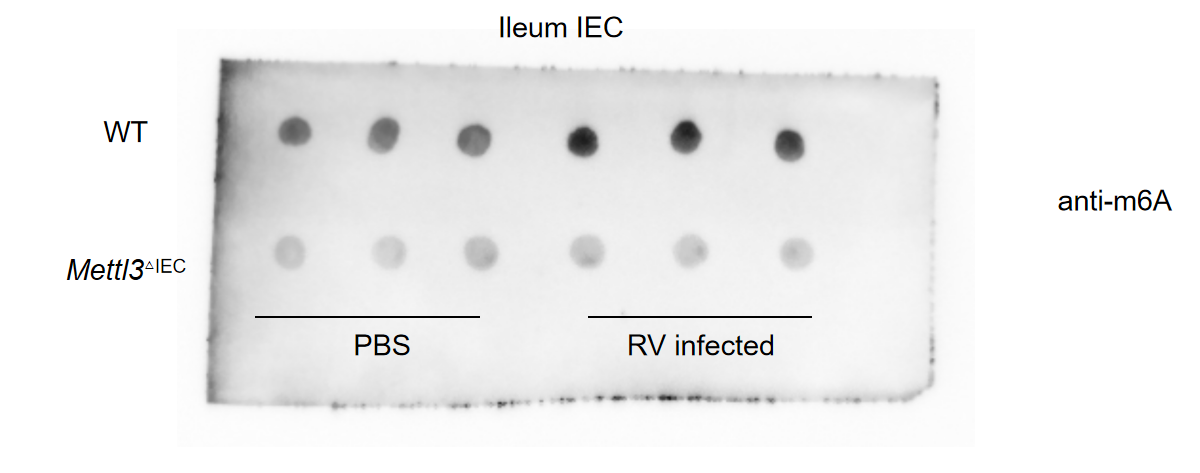

Supplement: Source data 1. [file elife-73628-data1.zip › Figure/Source data to Figure1/figure 1e.png]

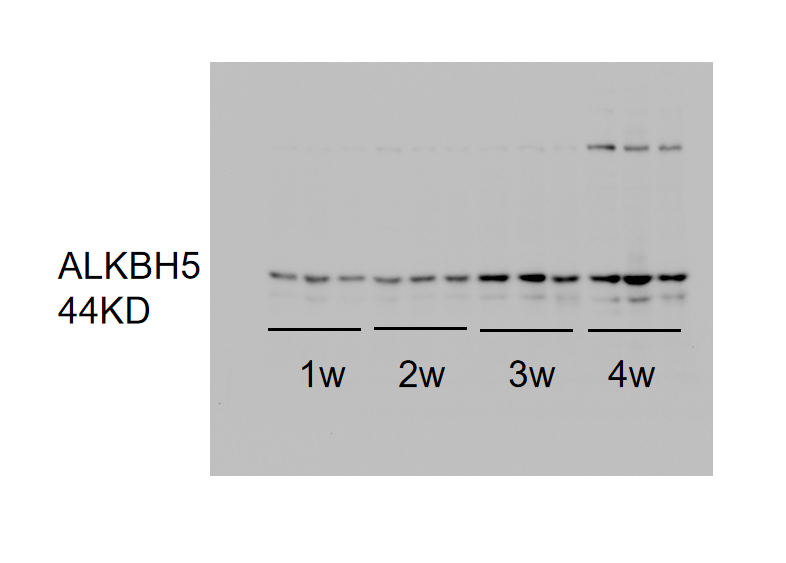

Supplement: Source data 1. [file elife-73628-data1.zip › Figure/Source data to Figure1 sup-figure 1/sup fig 1a-ALKBH5.png]

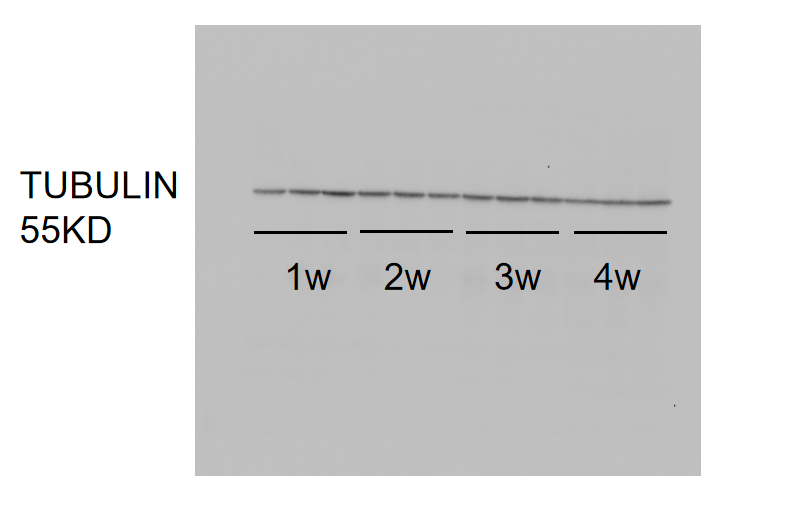

Supplement: Source data 1. [file elife-73628-data1.zip › Figure/Source data to Figure1 sup-figure 1/sup fig 1a-TUBULIN.png]

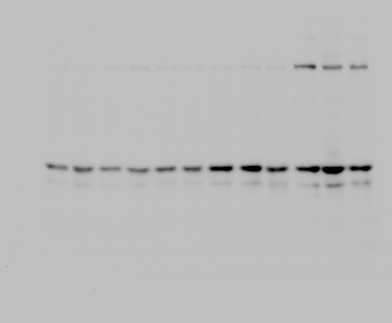

Supplement: Source data 1. [file elife-73628-data1.zip › Figure/Source data to Figure1 sup-figure 1/sup fig 1a-raw-ALKBH5.png]

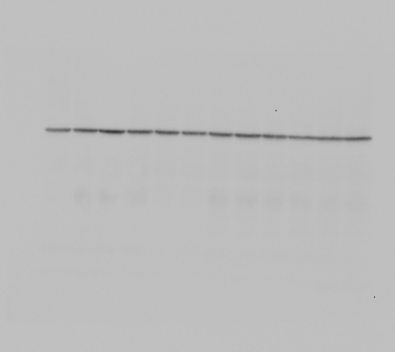

Supplement: Source data 1. [file elife-73628-data1.zip › Figure/Source data to Figure1 sup-figure 1/sup fig 1a-raw-TUBULIN.png]

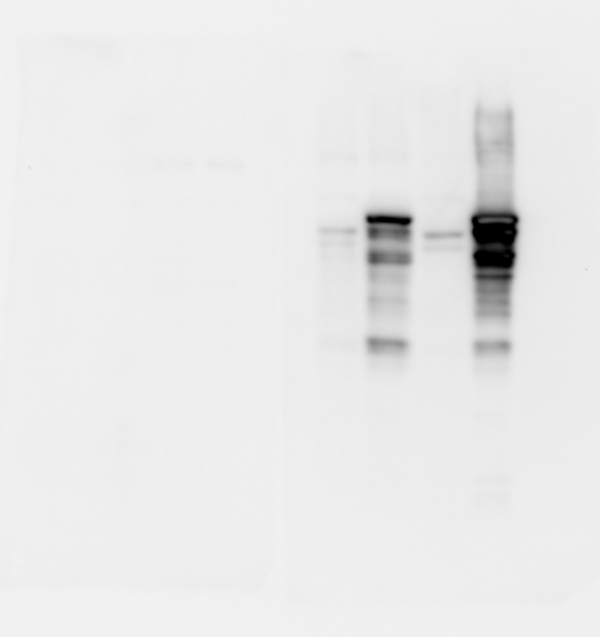

Supplement: Source data 1. [file elife-73628-data1.zip › Figure/Source data to Figure1 sup-figure 1/sup fig 1c-ALKBH5-raw.png]

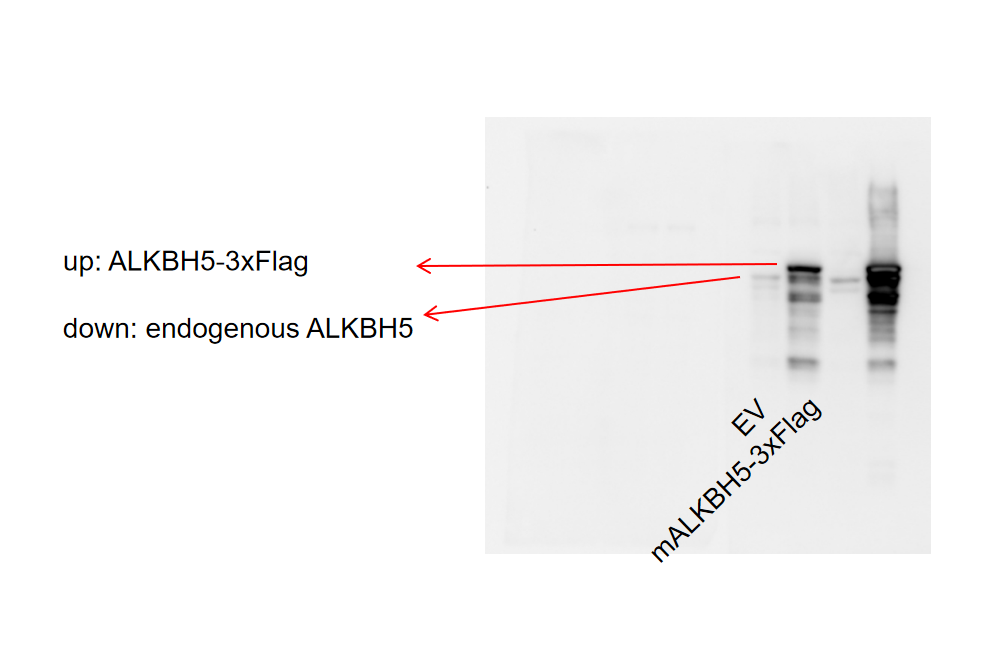

Supplement: Source data 1. [file elife-73628-data1.zip › Figure/Source data to Figure1 sup-figure 1/sup fig 1c-ALKBH5.png]

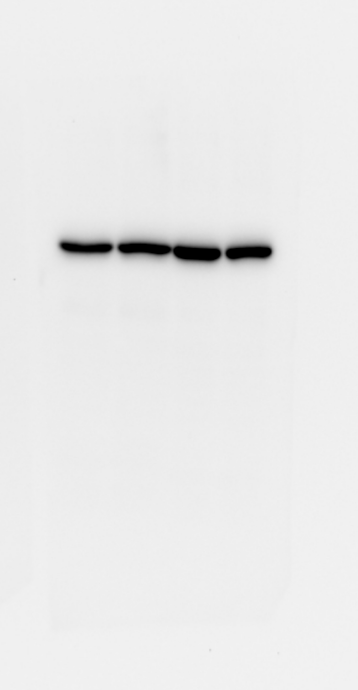

Supplement: Source data 1. [file elife-73628-data1.zip › Figure/Source data to Figure1 sup-figure 1/sup fig 1c-TUBULIN-raw.png]

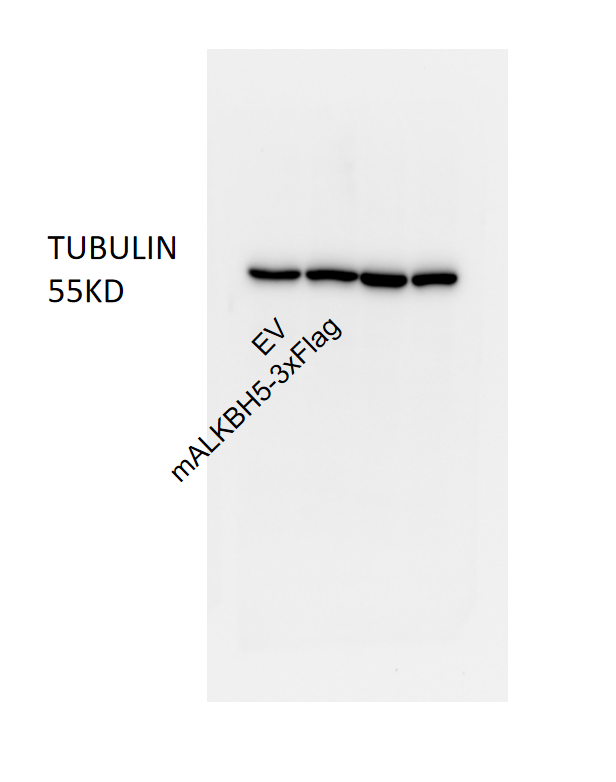

Supplement: Source data 1. [file elife-73628-data1.zip › Figure/Source data to Figure1 sup-figure 1/sup fig 1c-TUBULIN.png]

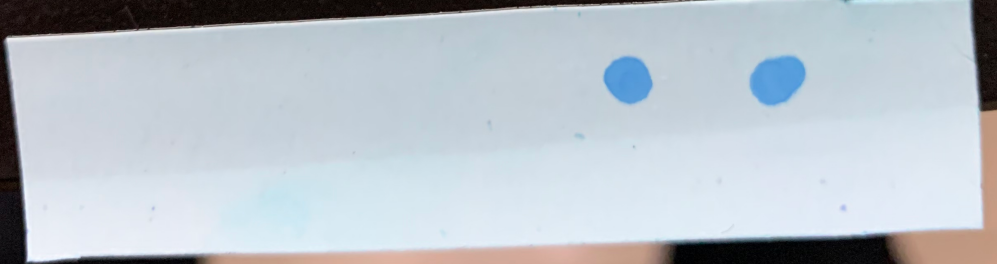

Supplement: Source data 1. [file elife-73628-data1.zip › Figure/Source data to Figure1 sup-figure 1/sup fig 1c-m6A-methyl-raw.png]

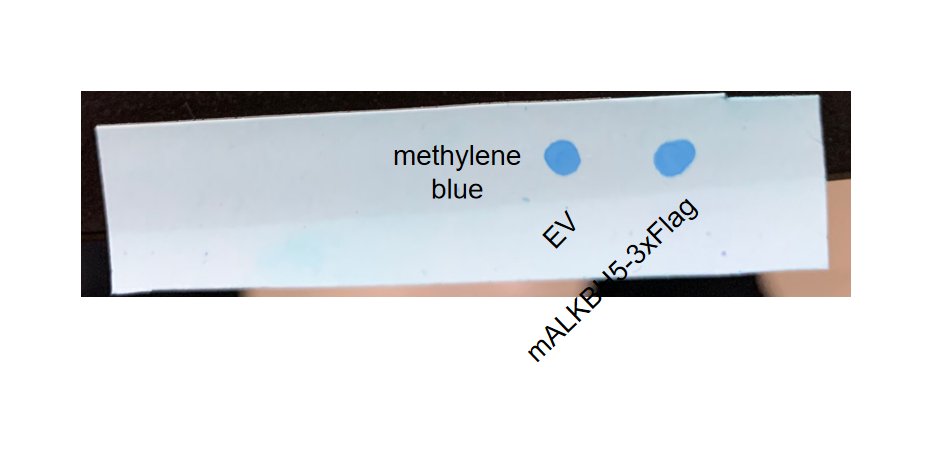

Supplement: Source data 1. [file elife-73628-data1.zip › Figure/Source data to Figure1 sup-figure 1/sup fig 1c-m6A-methyl.png]

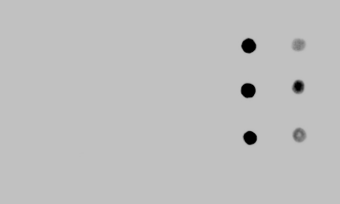

Supplement: Source data 1. [file elife-73628-data1.zip › Figure/Source data to Figure1 sup-figure 1/sup fig 1c-m6A-raw.png]

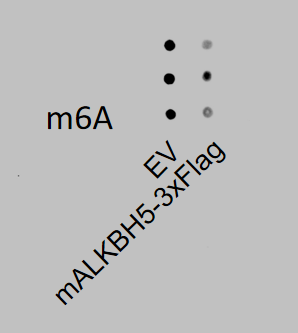

Supplement: Source data 1. [file elife-73628-data1.zip › Figure/Source data to Figure1 sup-figure 1/sup fig 1c-m6A.png]

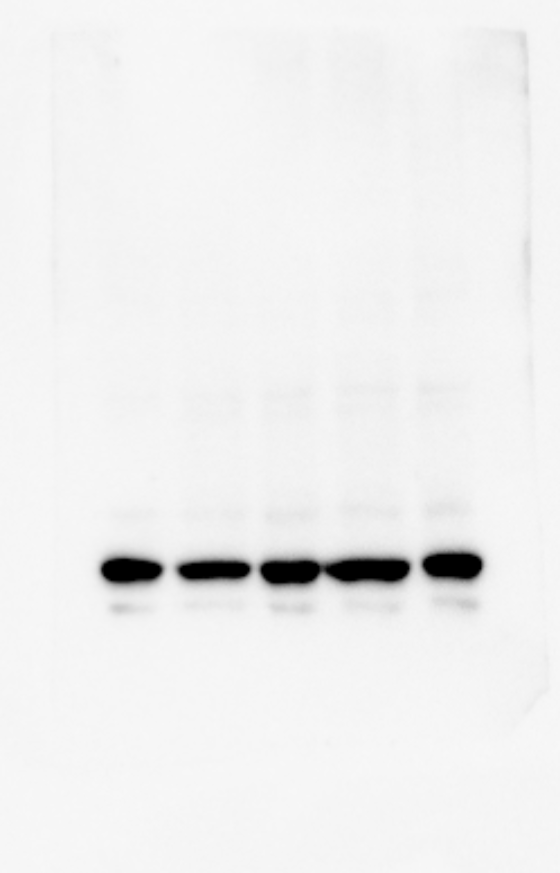

Supplement: Source data 1. [file elife-73628-data1.zip › Figure/Source data to Figure4 sup-figure 1/sup fig 1b-ACTIN-raw.tif]

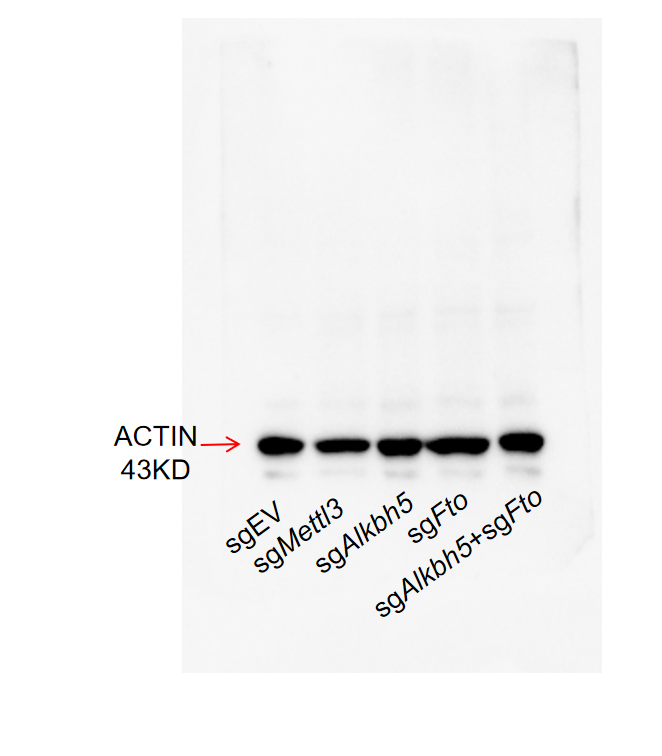

Supplement: Source data 1. [file elife-73628-data1.zip › Figure/Source data to Figure4 sup-figure 1/sup fig 1b-ACTIN.png]

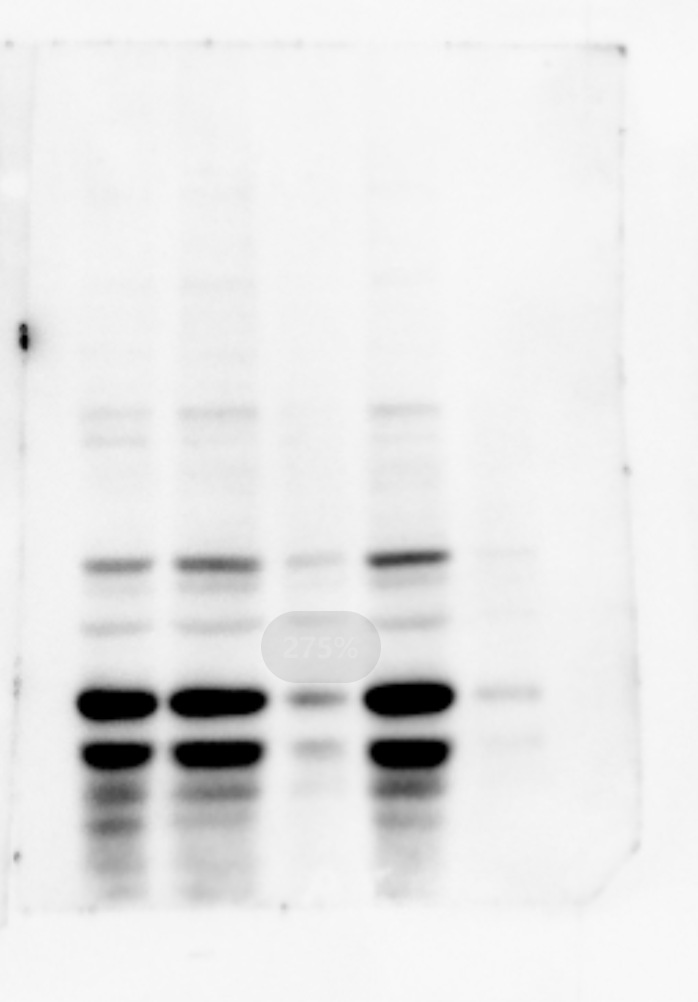

Supplement: Source data 1. [file elife-73628-data1.zip › Figure/Source data to Figure4 sup-figure 1/sup fig 1b-ALKBH5-raw.tif]

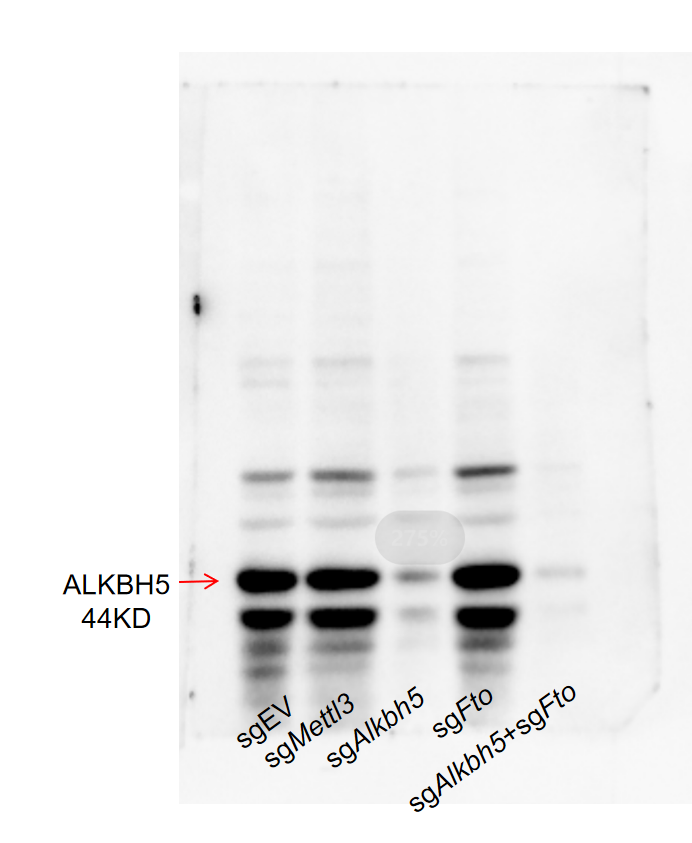

Supplement: Source data 1. [file elife-73628-data1.zip › Figure/Source data to Figure4 sup-figure 1/sup fig 1b-ALKBH5.png]

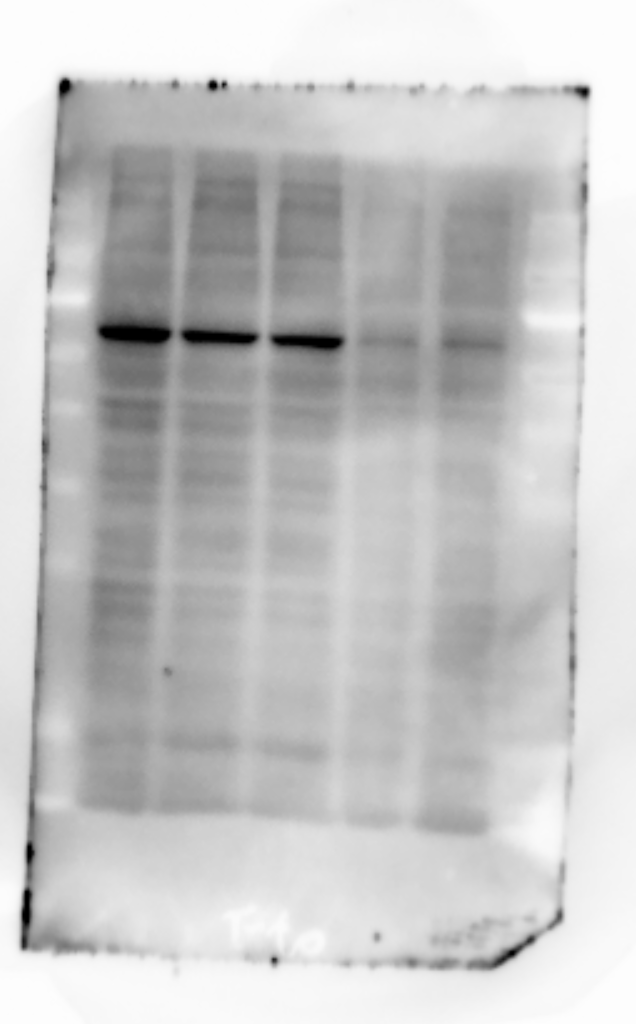

Supplement: Source data 1. [file elife-73628-data1.zip › Figure/Source data to Figure4 sup-figure 1/sup fig 1b-FTO-raw.tif]

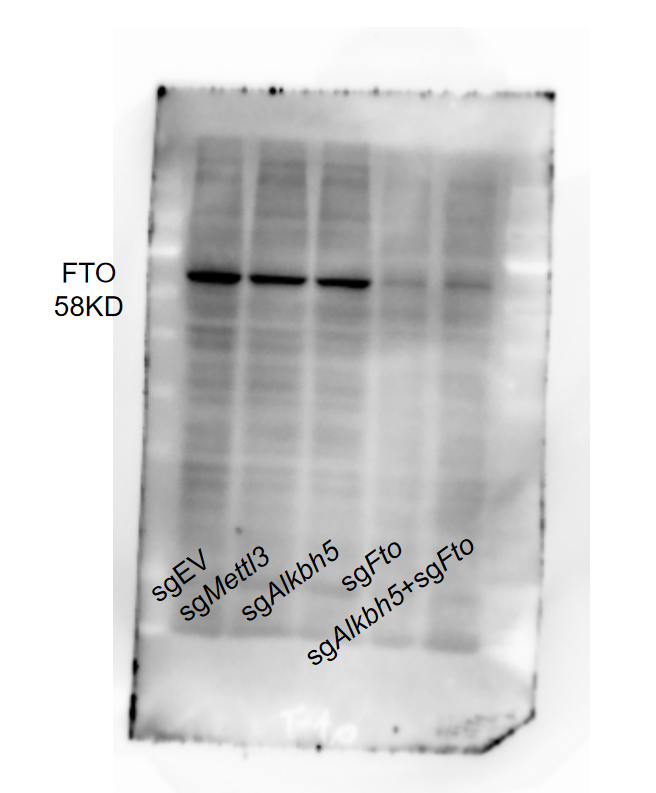

Supplement: Source data 1. [file elife-73628-data1.zip › Figure/Source data to Figure4 sup-figure 1/sup fig 1b-FTO.png]

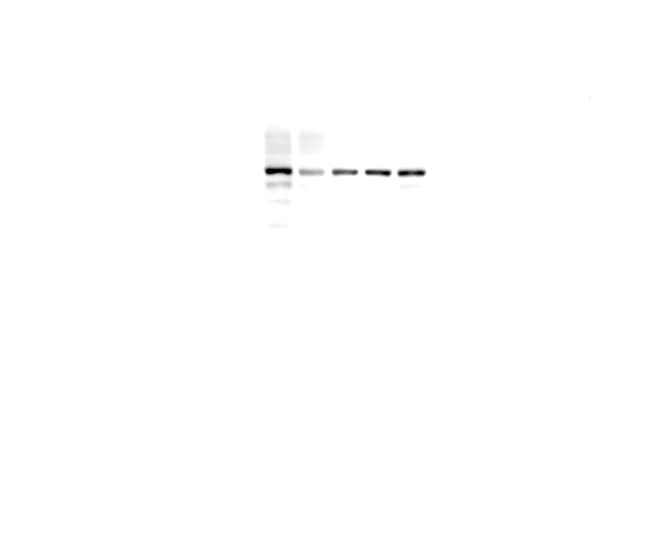

Supplement: Source data 1. [file elife-73628-data1.zip › Figure/Source data to Figure4 sup-figure 1/sup fig 1b-METTL3-raw.tif]

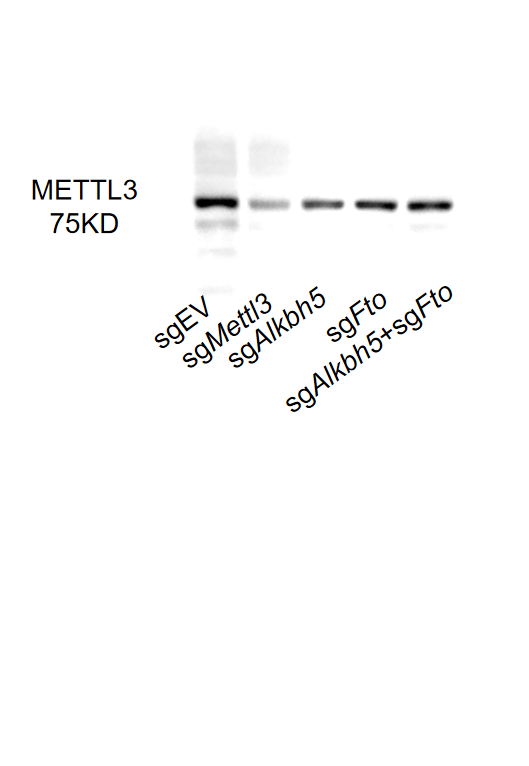

Supplement: Source data 1. [file elife-73628-data1.zip › Figure/Source data to Figure4 sup-figure 1/sup fig 1b-METTL3.png]

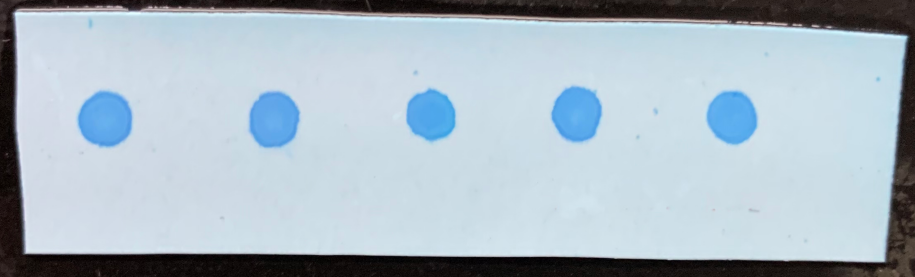

Supplement: Source data 1. [file elife-73628-data1.zip › Figure/Source data to Figure4 sup-figure 1/sup fig 1b-m6A-methyl-raw.tif]

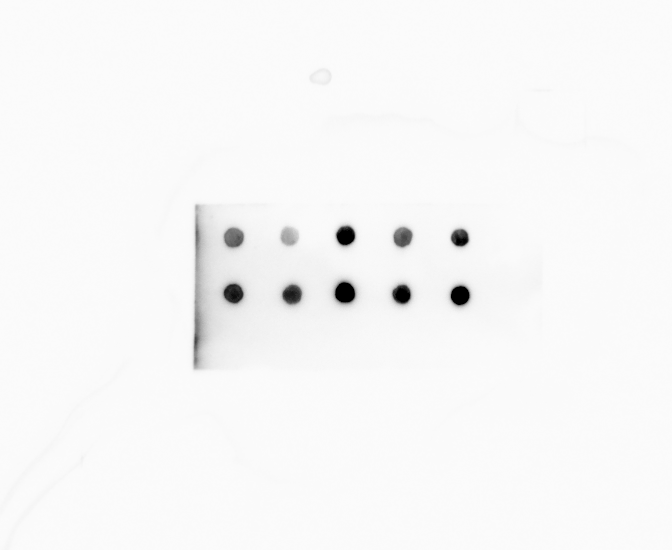

Supplement: Source data 1. [file elife-73628-data1.zip › Figure/Source data to Figure4 sup-figure 1/sup fig 1b-m6A-raw.tif]

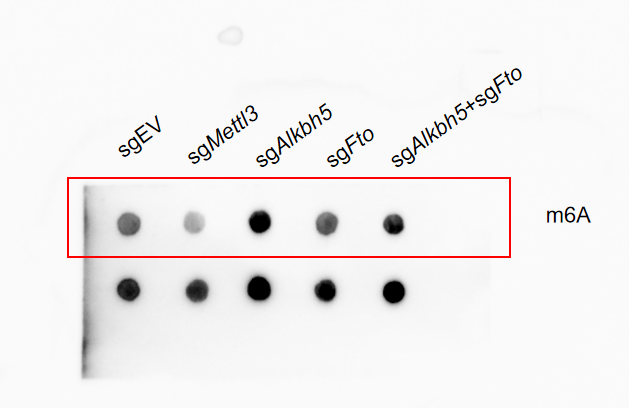

Supplement: Source data 1. [file elife-73628-data1.zip › Figure/Source data to Figure4 sup-figure 1/sup fig 1b-m6A.png]

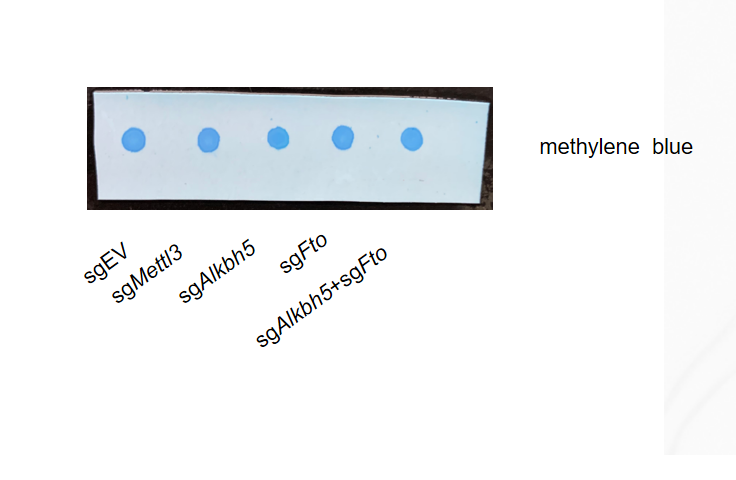

Supplement: Source data 1. [file elife-73628-data1.zip › Figure/Source data to Figure4 sup-figure 1/sup fig 1b-methyl.png]

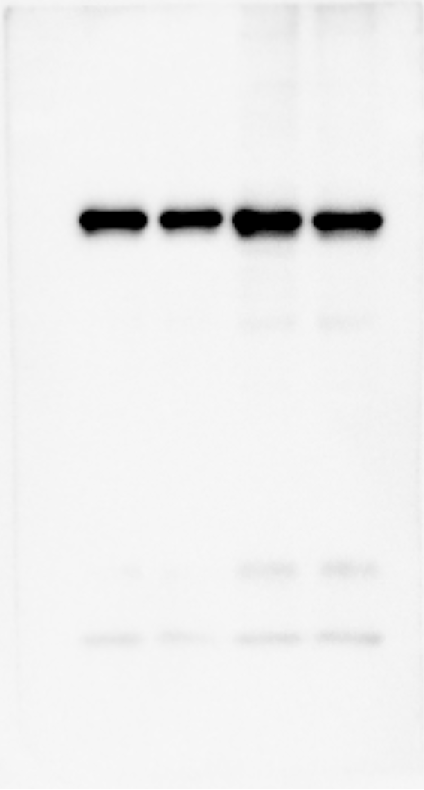

Supplement: Source data 1. [file elife-73628-data1.zip › Figure/Source data to Figure4 sup-figure 2/sup fig 2b-ACTIN-raw.png]

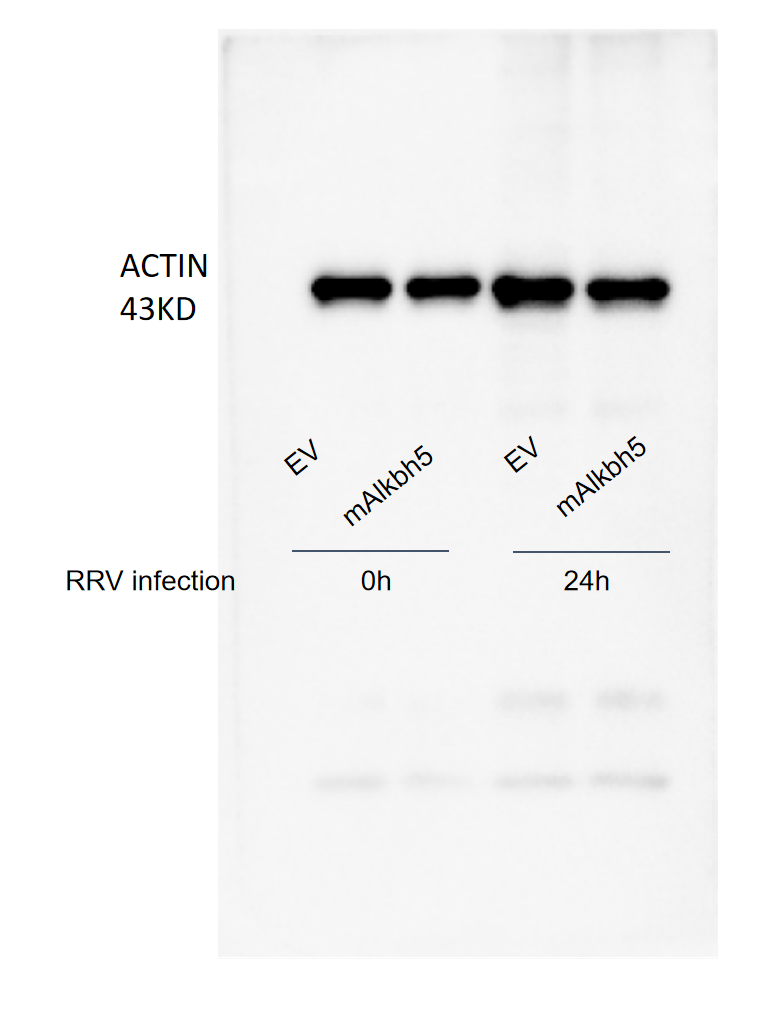

Supplement: Source data 1. [file elife-73628-data1.zip › Figure/Source data to Figure4 sup-figure 2/sup fig 2b-ACTIN.png]

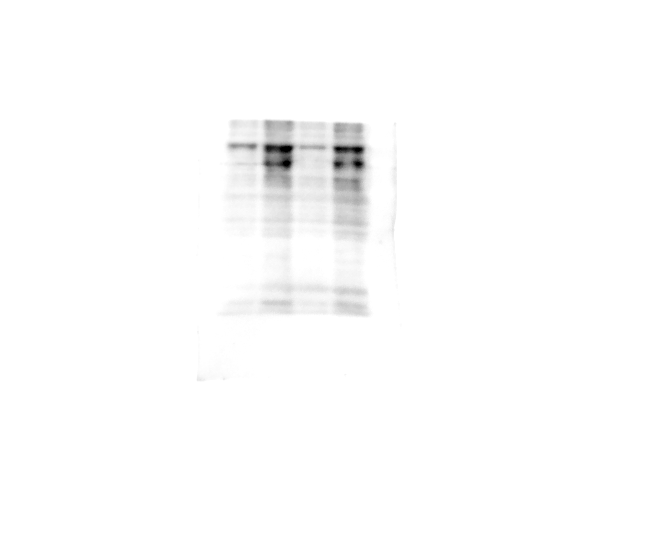

Supplement: Source data 1. [file elife-73628-data1.zip › Figure/Source data to Figure4 sup-figure 2/sup fig 2b-ALKBH5-raw.tif]

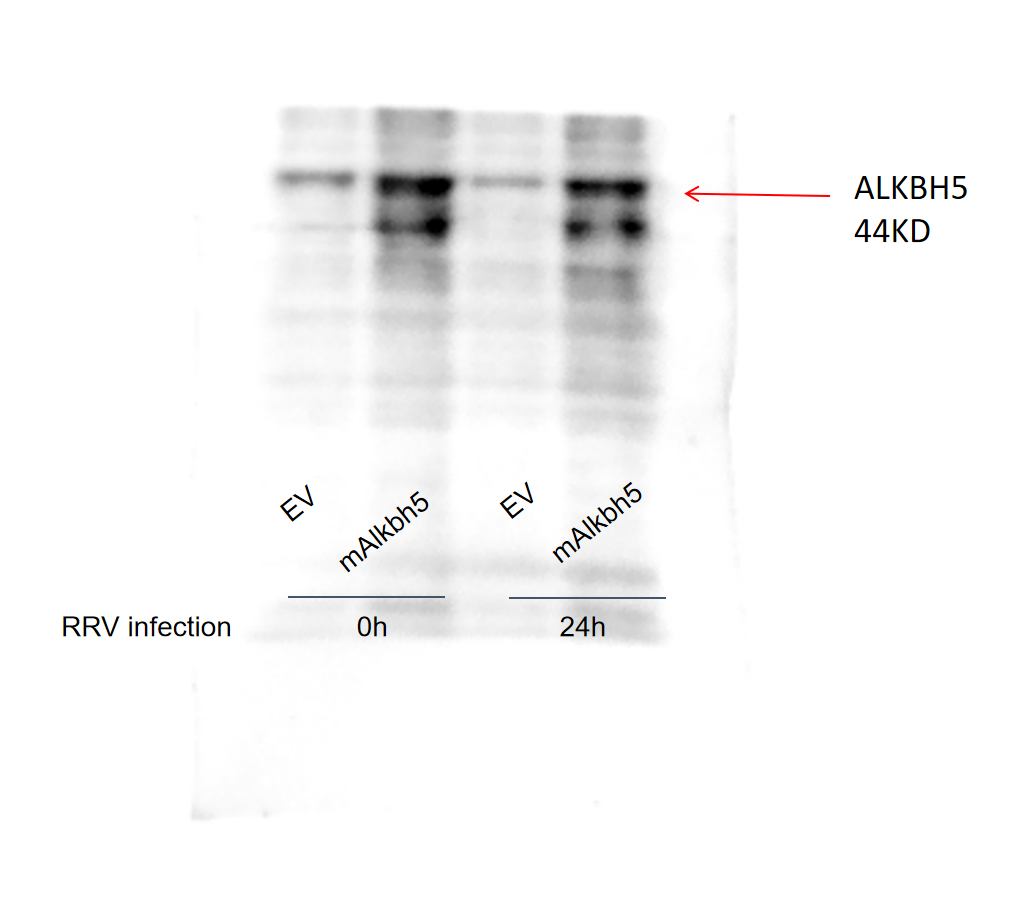

Supplement: Source data 1. [file elife-73628-data1.zip › Figure/Source data to Figure4 sup-figure 2/sup fig 2b-ALKBH5.png]

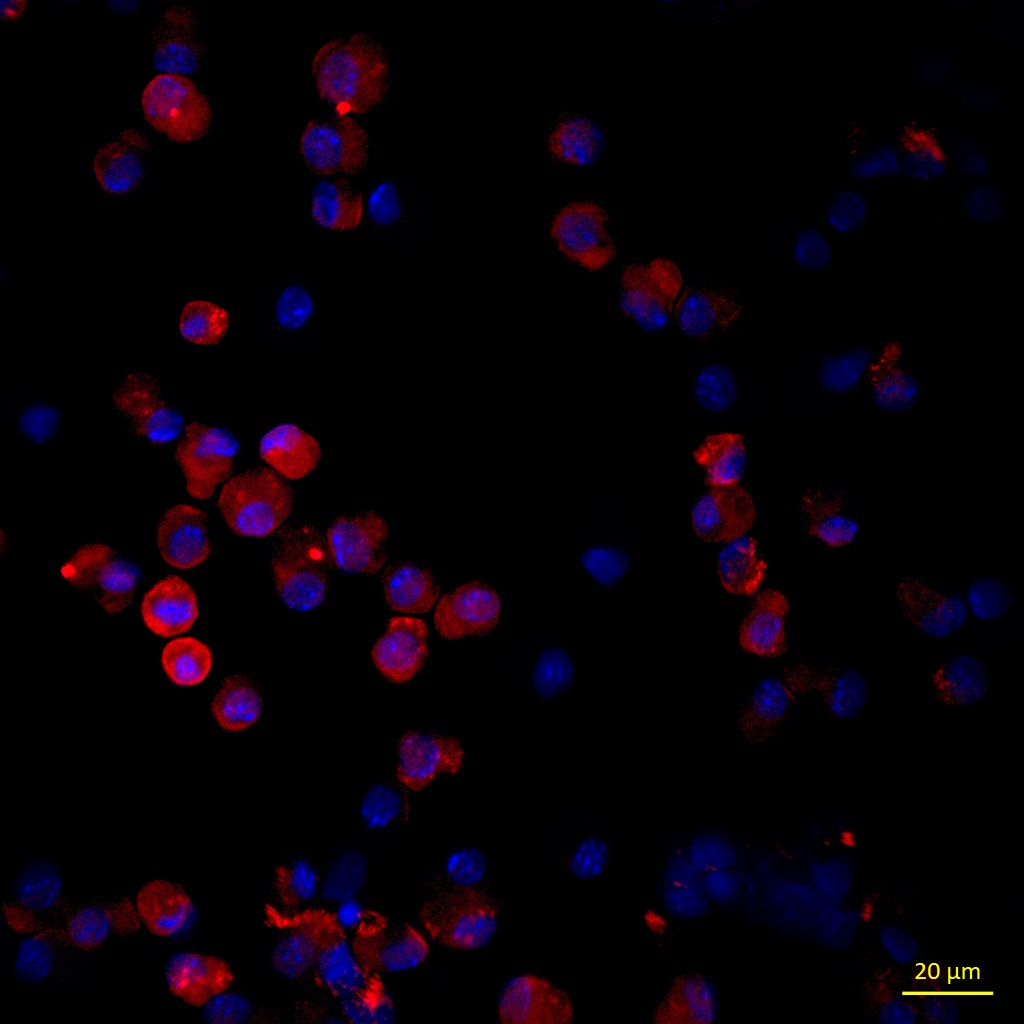

Supplement: Source data 1. [file elife-73628-data1.zip › Figure/Source data to Figure4 sup-figure 4/2020.6.23-m3ko-iec-J2/2020.6.23-m3ko-iec-J2-8_c1+2.jpg]

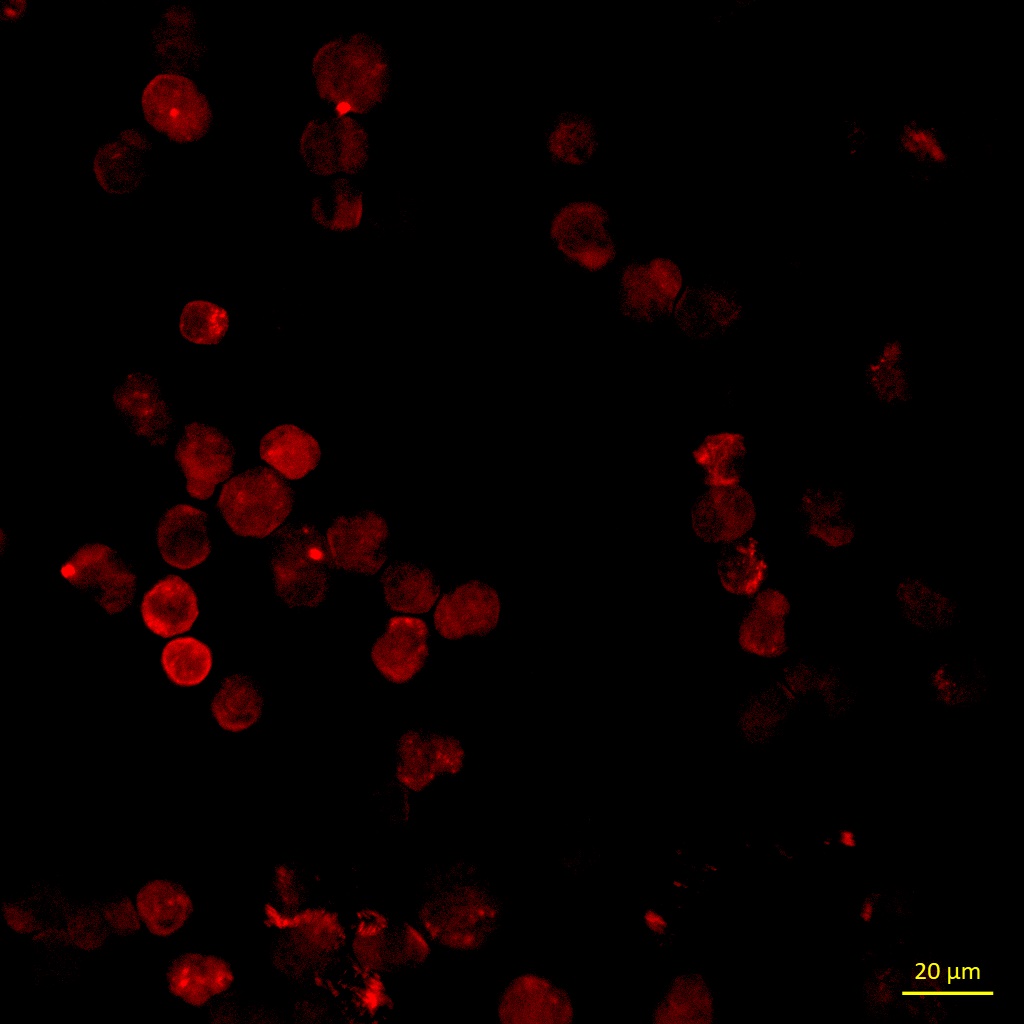

Supplement: Source data 1. [file elife-73628-data1.zip › Figure/Source data to Figure4 sup-figure 4/2020.6.23-m3ko-iec-J2/2020.6.23-m3ko-iec-J2-8_c1.jpg]

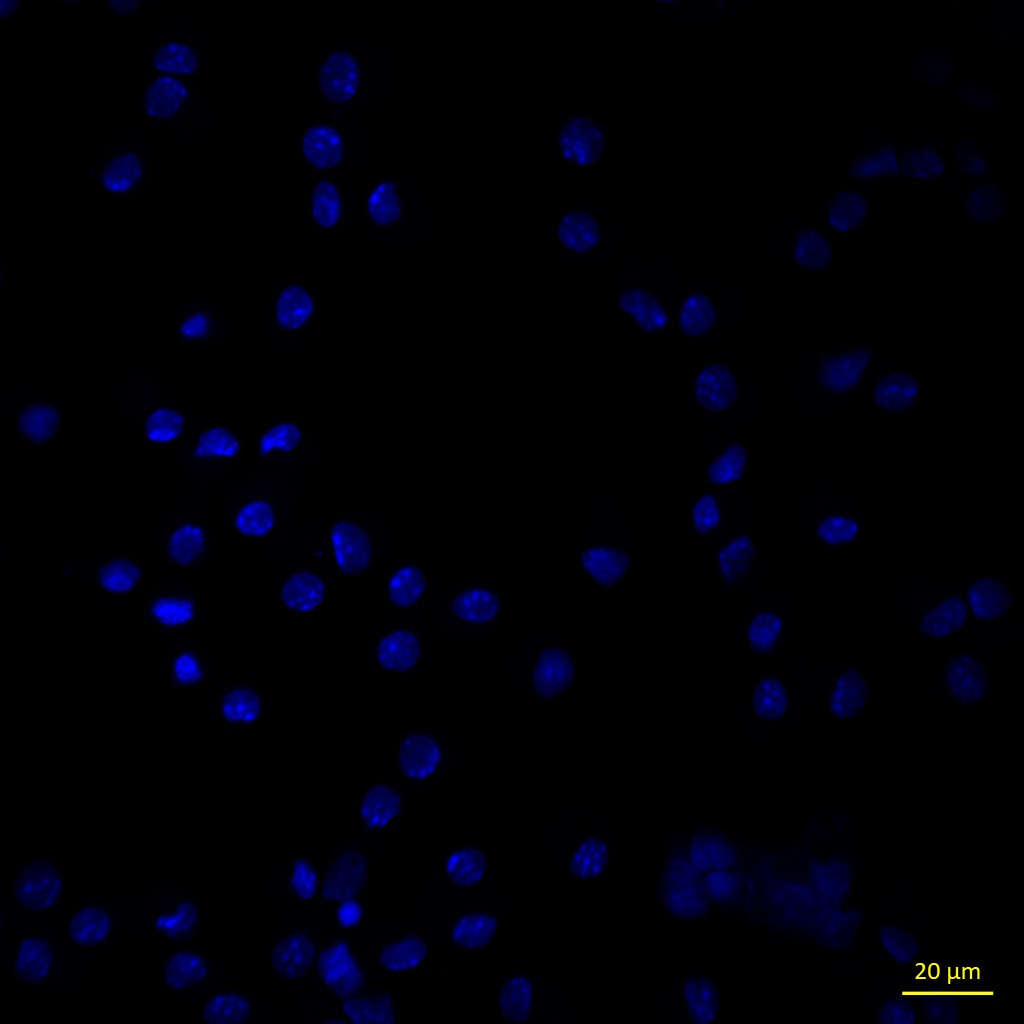

Supplement: Source data 1. [file elife-73628-data1.zip › Figure/Source data to Figure4 sup-figure 4/2020.6.23-m3ko-iec-J2/2020.6.23-m3ko-iec-J2-8_c2.jpg]

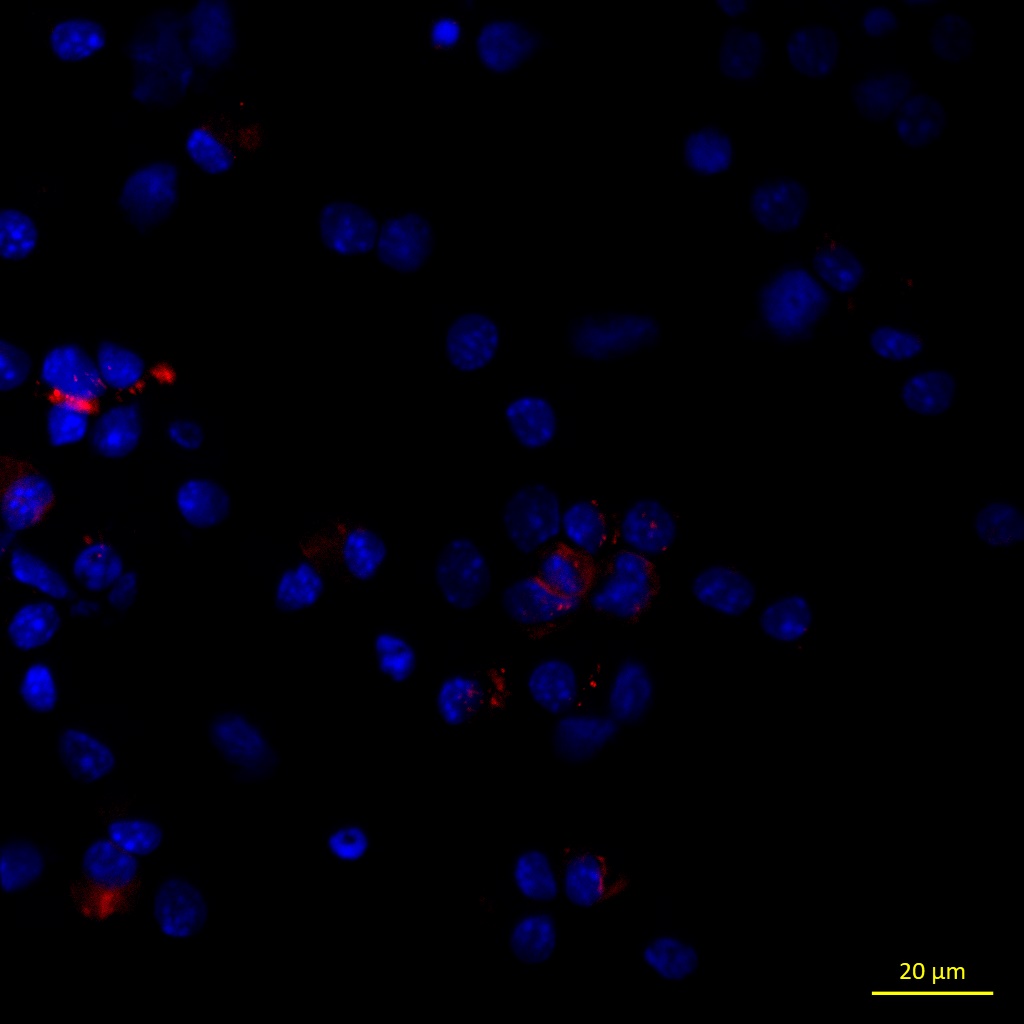

Supplement: Source data 1. [file elife-73628-data1.zip › Figure/Source data to Figure4 sup-figure 4/2020.6.23-m3wt-iec-J2/2020.6.23-m3wt-iec-J27_c1+2.jpg]

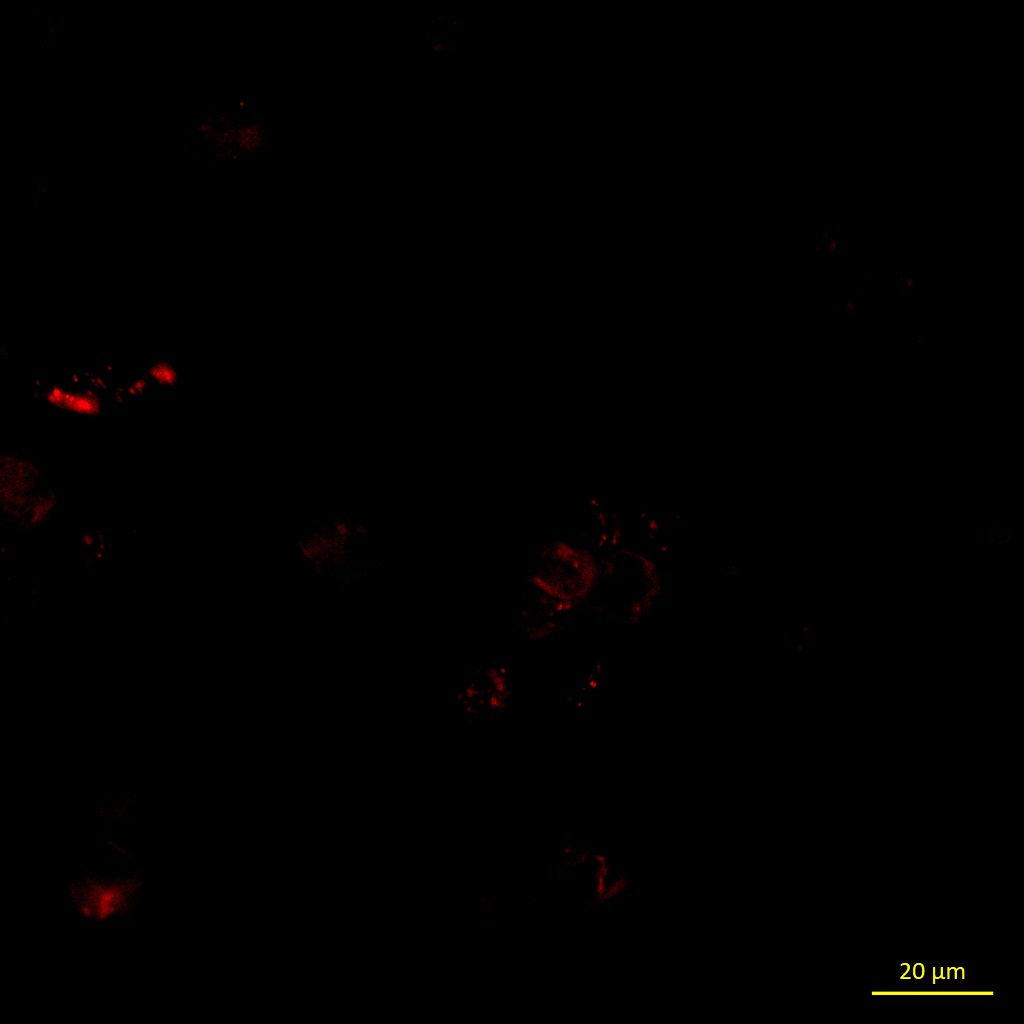

Supplement: Source data 1. [file elife-73628-data1.zip › Figure/Source data to Figure4 sup-figure 4/2020.6.23-m3wt-iec-J2/2020.6.23-m3wt-iec-J27_c1.jpg]

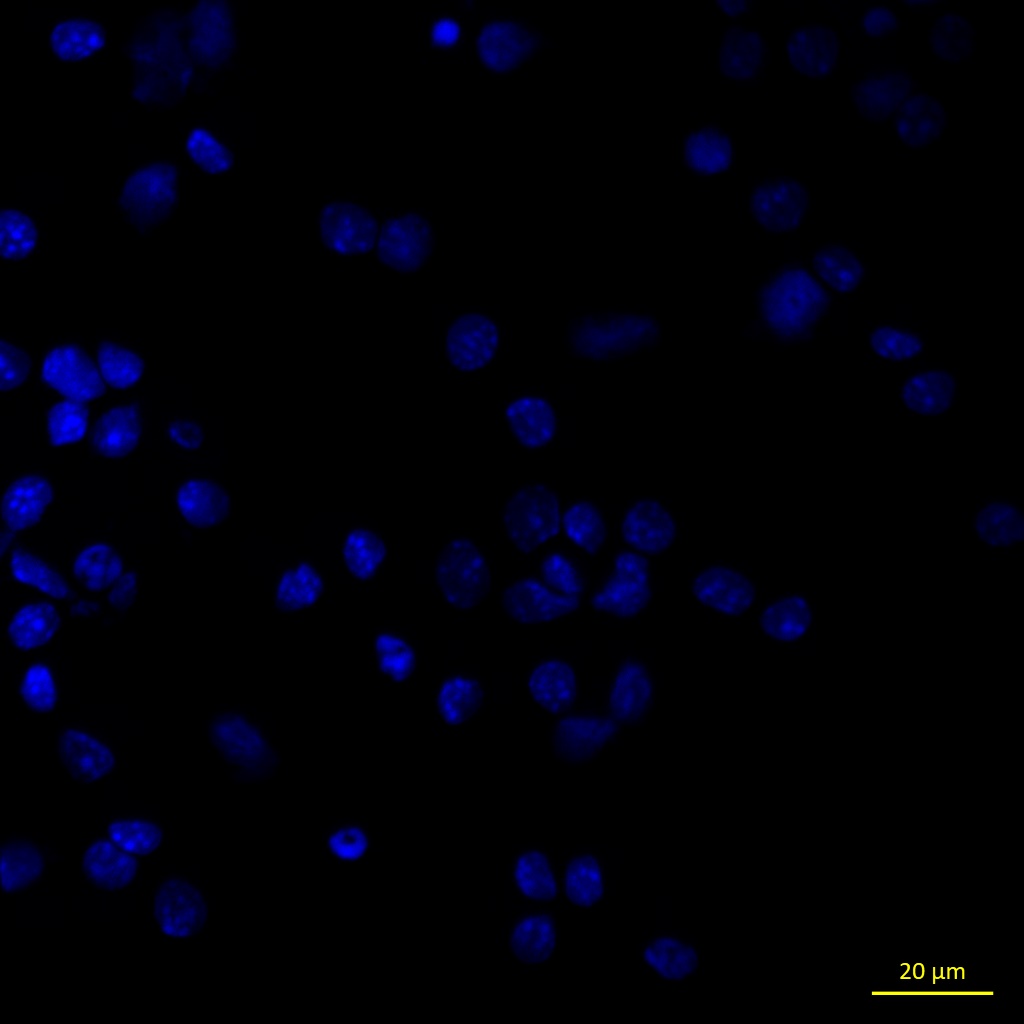

Supplement: Source data 1. [file elife-73628-data1.zip › Figure/Source data to Figure4 sup-figure 4/2020.6.23-m3wt-iec-J2/2020.6.23-m3wt-iec-J27_c2.jpg]

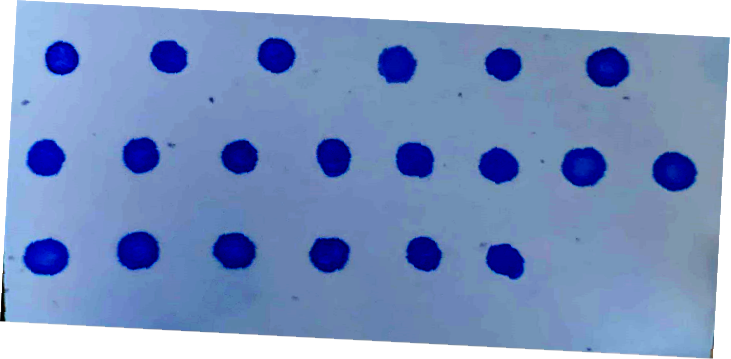

Supplement: Source data 1. [file elife-73628-data1.zip › Figure/Source data to Figure1 sup-figure 2/sup-fig 2a-m6A-methyl-raw.png]

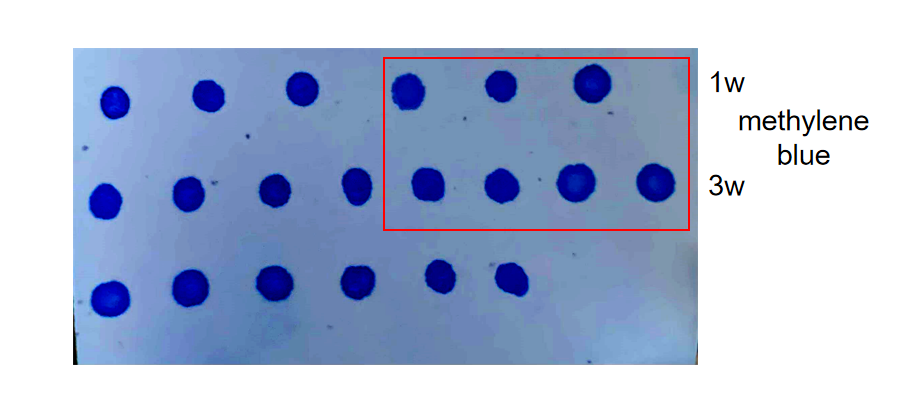

Supplement: Source data 1. [file elife-73628-data1.zip › Figure/Source data to Figure1 sup-figure 2/sup-fig 2a-m6A-methyl.png]

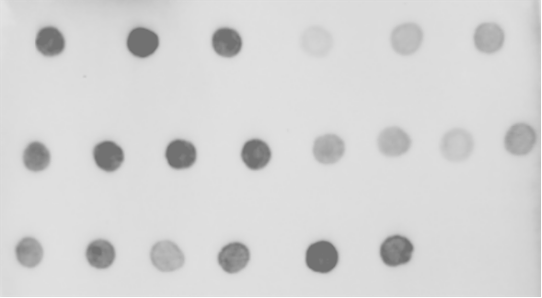

Supplement: Source data 1. [file elife-73628-data1.zip › Figure/Source data to Figure1 sup-figure 2/sup-fig 2a-m6A-raw.png]

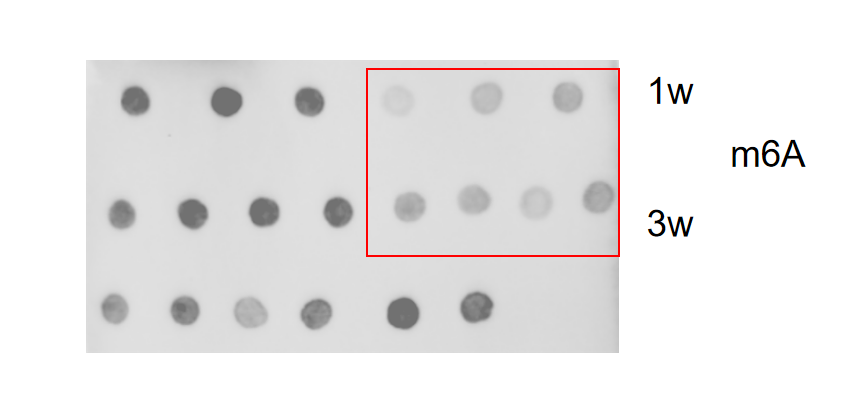

Supplement: Source data 1. [file elife-73628-data1.zip › Figure/Source data to Figure1 sup-figure 2/sup-fig 2a-m6A.png]

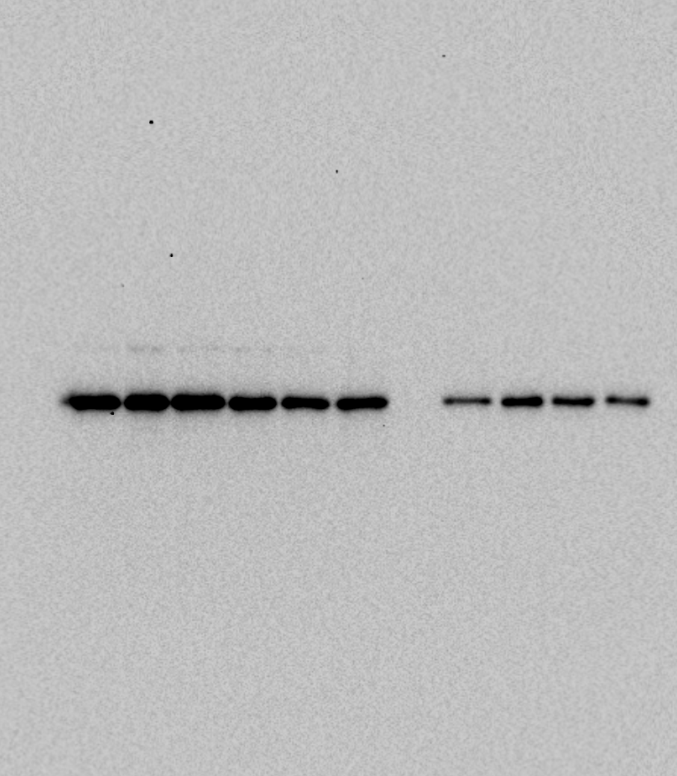

Supplement: Source data 1. [file elife-73628-data1.zip › Figure/Source data to Figure2 sup-figure 1/sup fig 1d-GAPDH-raw.png]

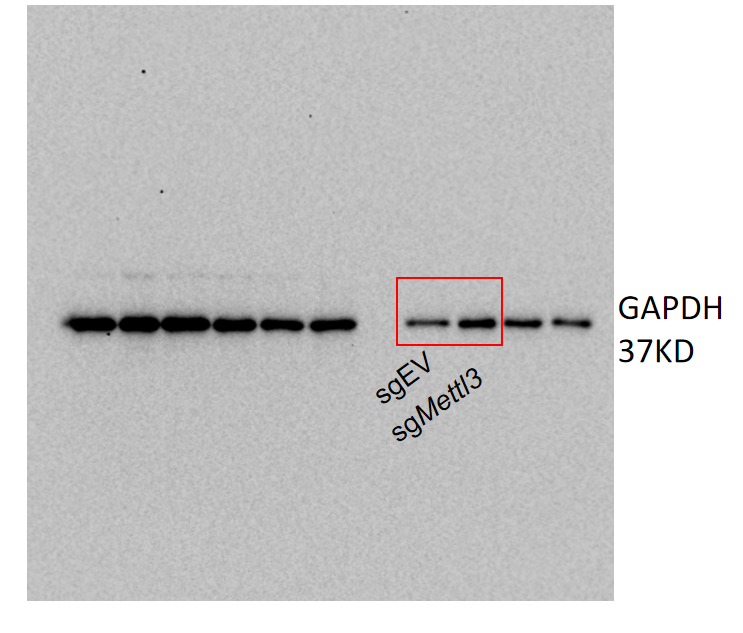

Supplement: Source data 1. [file elife-73628-data1.zip › Figure/Source data to Figure2 sup-figure 1/sup fig 1d-GAPDH.png]

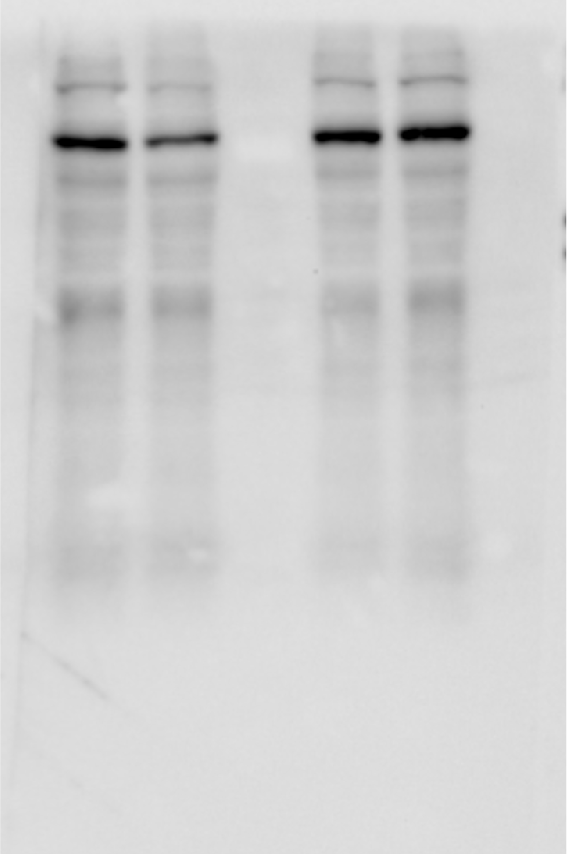

Supplement: Source data 1. [file elife-73628-data1.zip › Figure/Source data to Figure2 sup-figure 1/sup fig 1e-METTL3-raw.png]

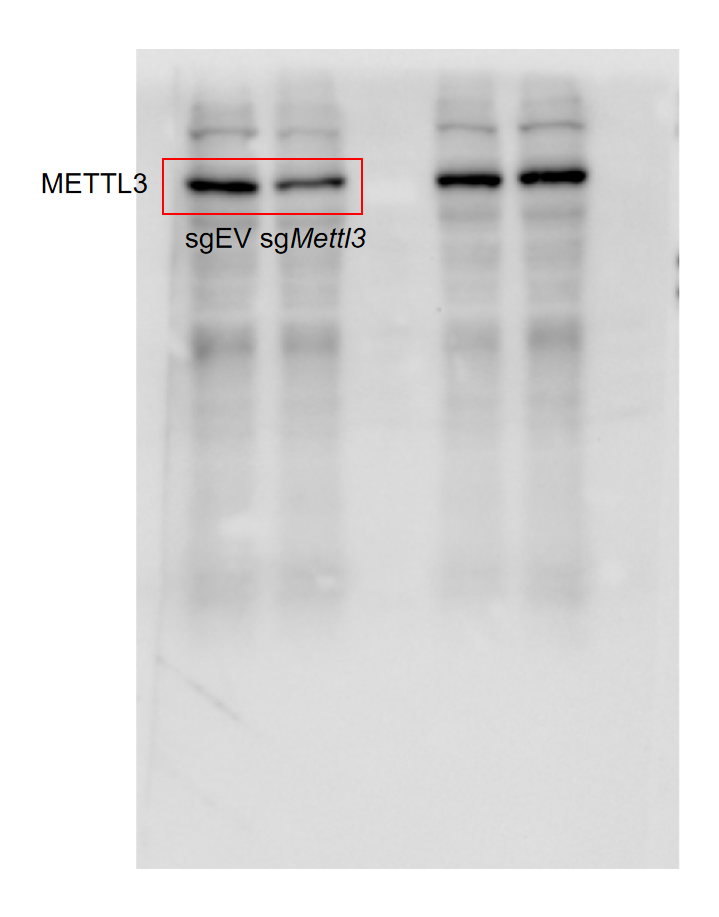

Supplement: Source data 1. [file elife-73628-data1.zip › Figure/Source data to Figure2 sup-figure 1/sup fig 1e-METTL3.png]

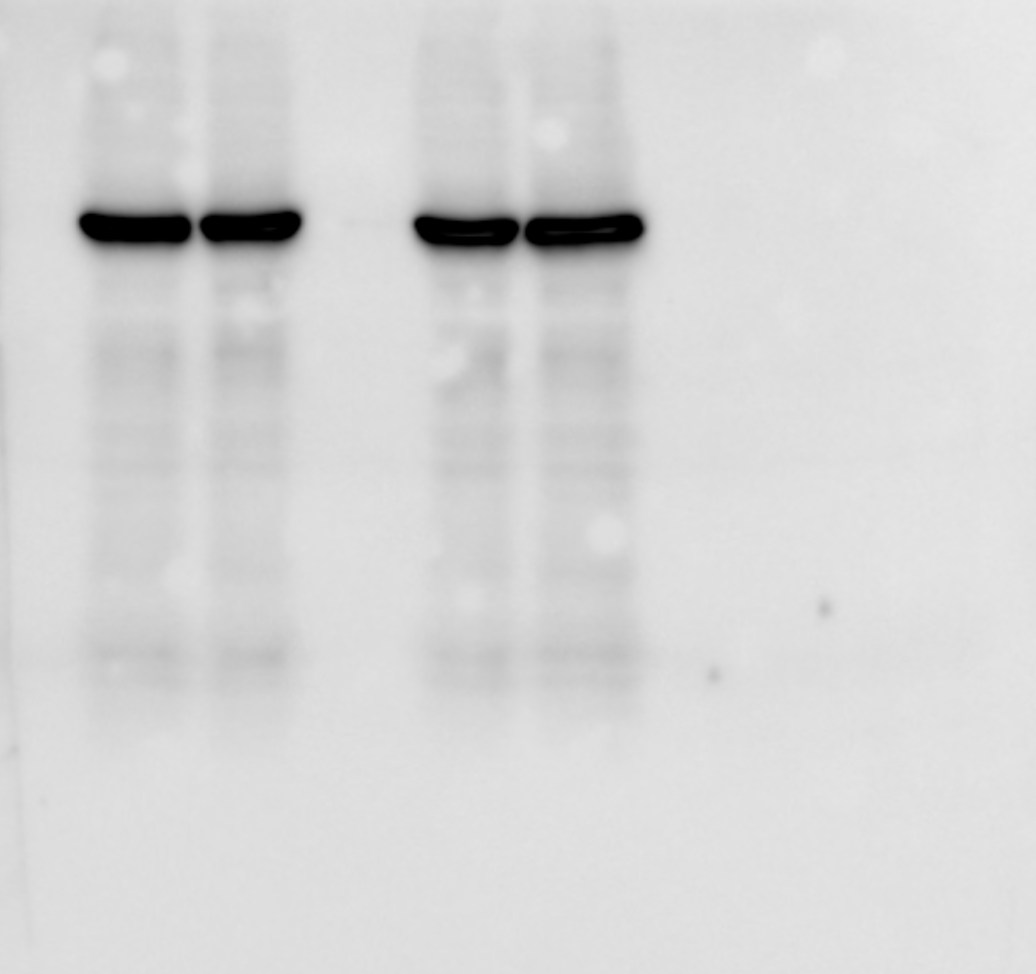

Supplement: Source data 1. [file elife-73628-data1.zip › Figure/Source data to Figure2 sup-figure 1/sup fig 1e-TUBULIN-raw.png]

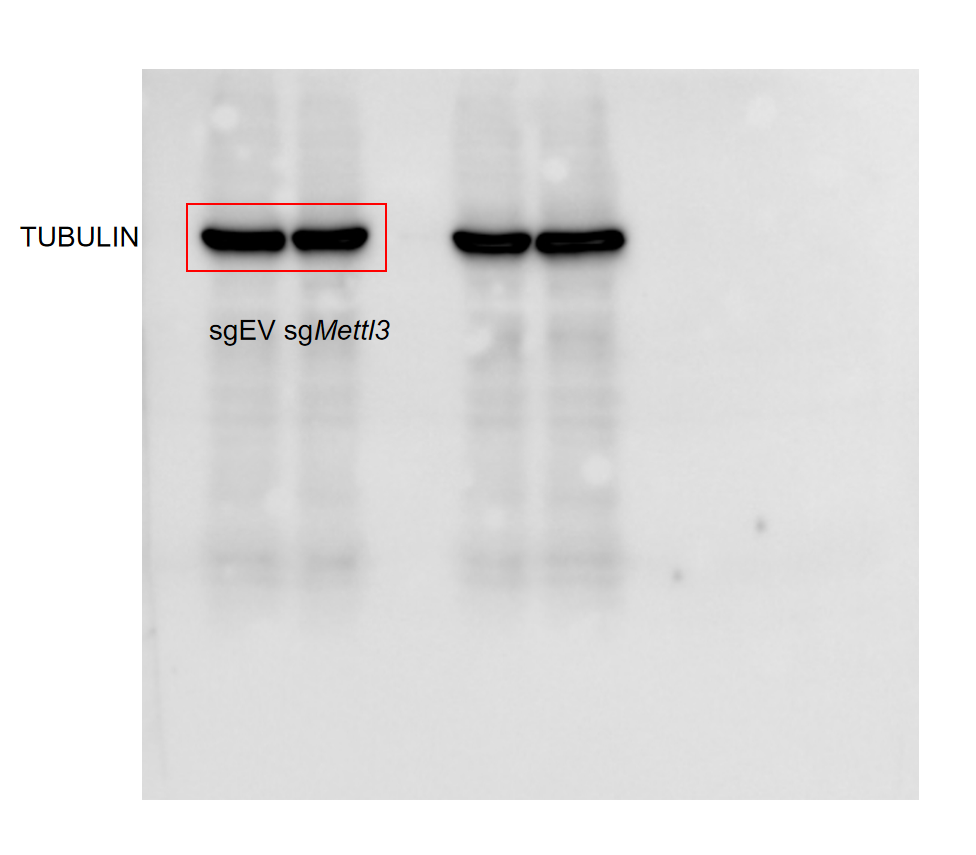

Supplement: Source data 1. [file elife-73628-data1.zip › Figure/Source data to Figure2 sup-figure 1/sup fig 1e-TUBULIN.png]

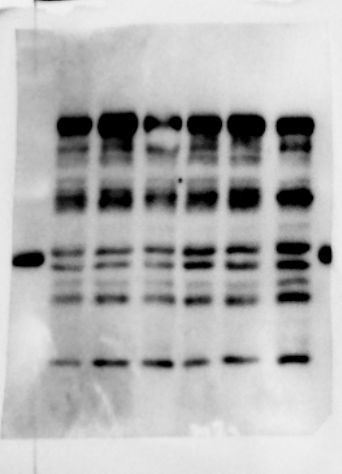

Supplement: Source data 1. [file elife-73628-data1.zip › Figure/Source data to Figure2 sup-figure 1/sup fig 1f/sup fig 5f-IRF7-raw.png]

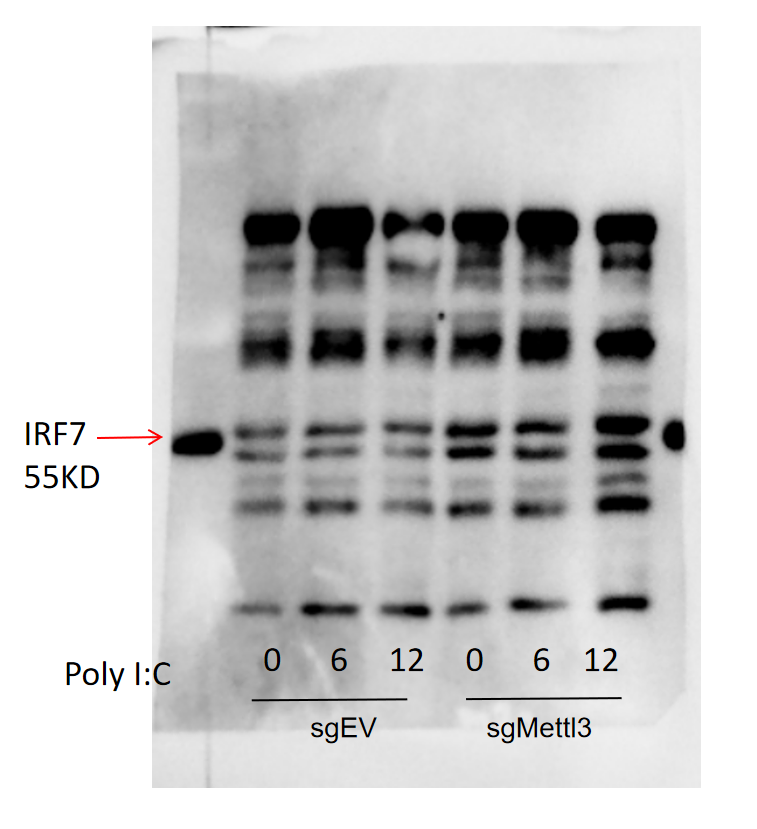

Supplement: Source data 1. [file elife-73628-data1.zip › Figure/Source data to Figure2 sup-figure 1/sup fig 1f/sup fig 5f-IRF7.png]

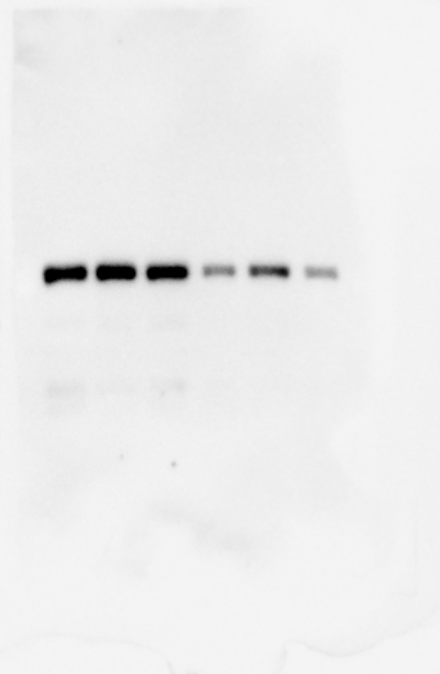

Supplement: Source data 1. [file elife-73628-data1.zip › Figure/Source data to Figure2 sup-figure 1/sup fig 1f/sup fig 5f-METTL3-raw.png]

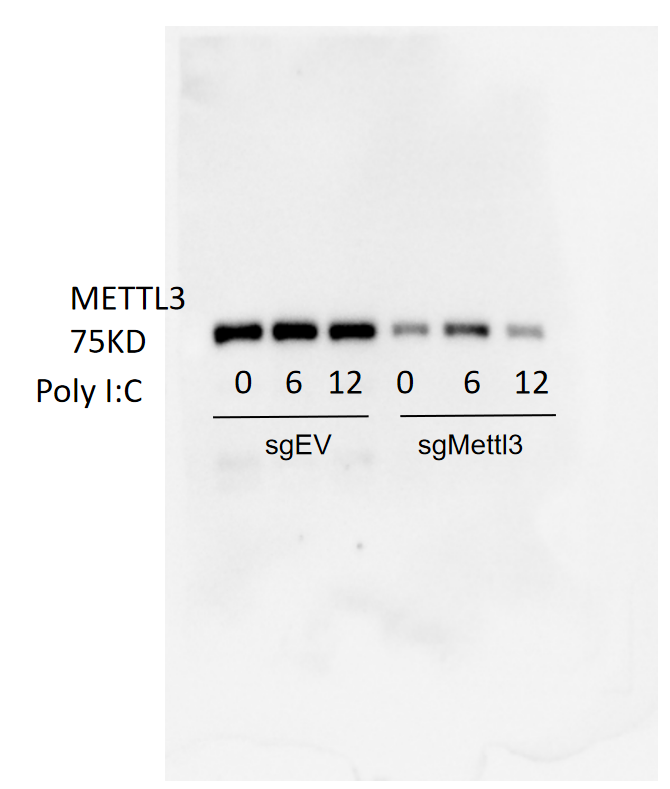

Supplement: Source data 1. [file elife-73628-data1.zip › Figure/Source data to Figure2 sup-figure 1/sup fig 1f/sup fig 5f-METTL3.png]

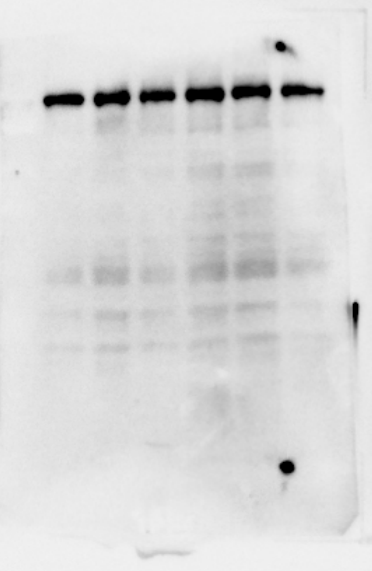

Supplement: Source data 1. [file elife-73628-data1.zip › Figure/Source data to Figure2 sup-figure 1/sup fig 1f/sup fig 5f-TBK1-raw.png]

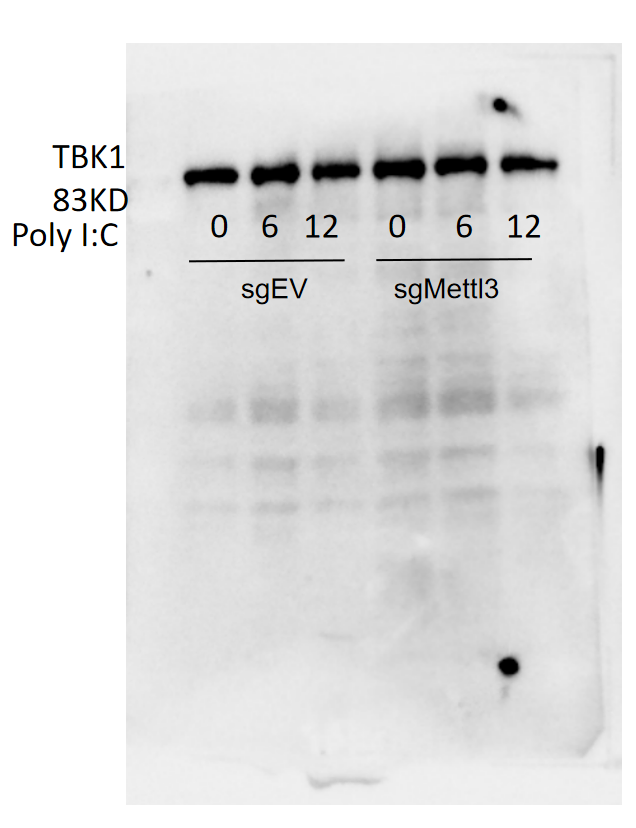

Supplement: Source data 1. [file elife-73628-data1.zip › Figure/Source data to Figure2 sup-figure 1/sup fig 1f/sup fig 5f-TBK1.png]

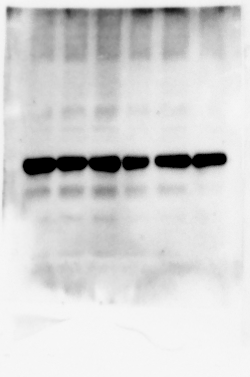

Supplement: Source data 1. [file elife-73628-data1.zip › Figure/Source data to Figure2 sup-figure 1/sup fig 1f/sup fig 5f-TUBULIN-raw.png]

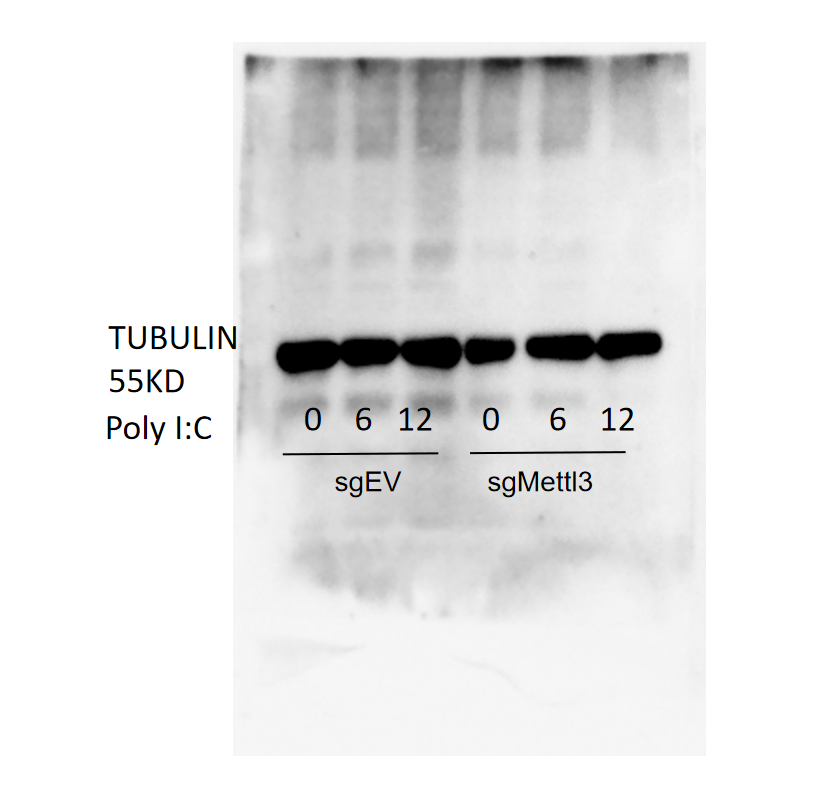

Supplement: Source data 1. [file elife-73628-data1.zip › Figure/Source data to Figure2 sup-figure 1/sup fig 1f/sup fig 5f-TUBULIN.png]

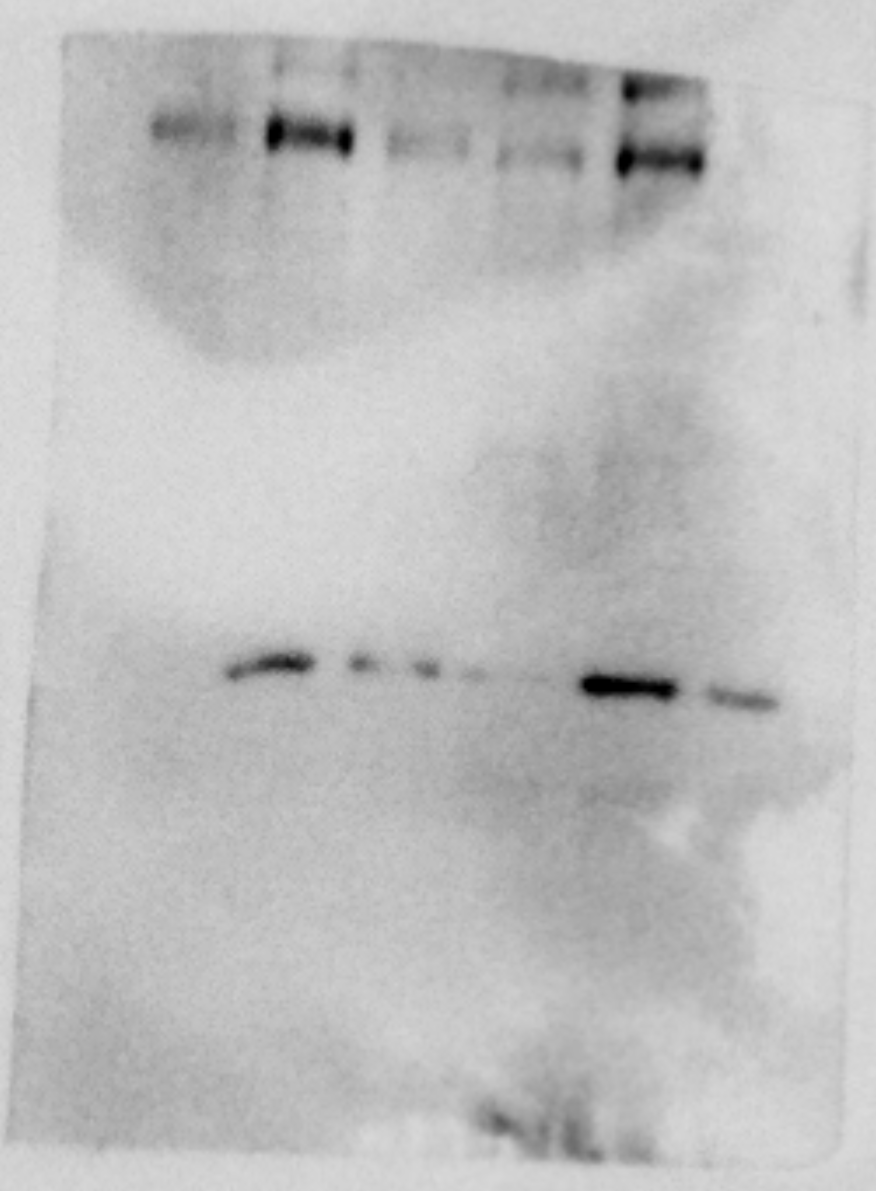

Supplement: Source data 1. [file elife-73628-data1.zip › Figure/Source data to Figure2 sup-figure 1/sup fig 1f/sup-fig 5f-p-IRF7-raw.png]

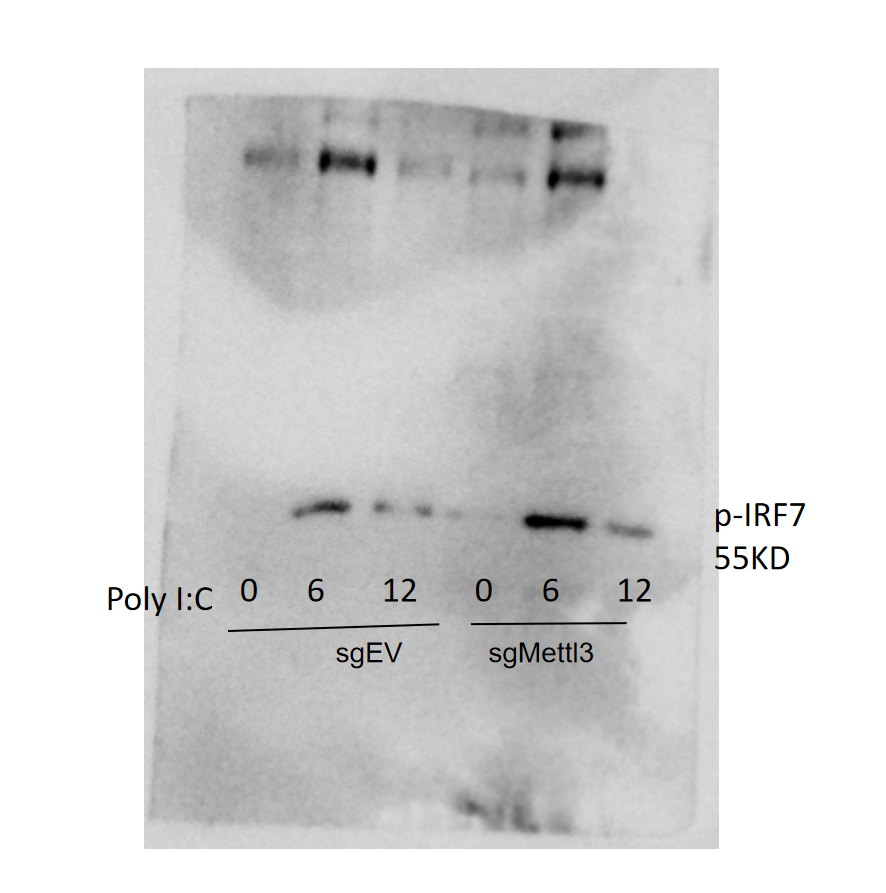

Supplement: Source data 1. [file elife-73628-data1.zip › Figure/Source data to Figure2 sup-figure 1/sup fig 1f/sup-fig 5f-p-IRF7.png]

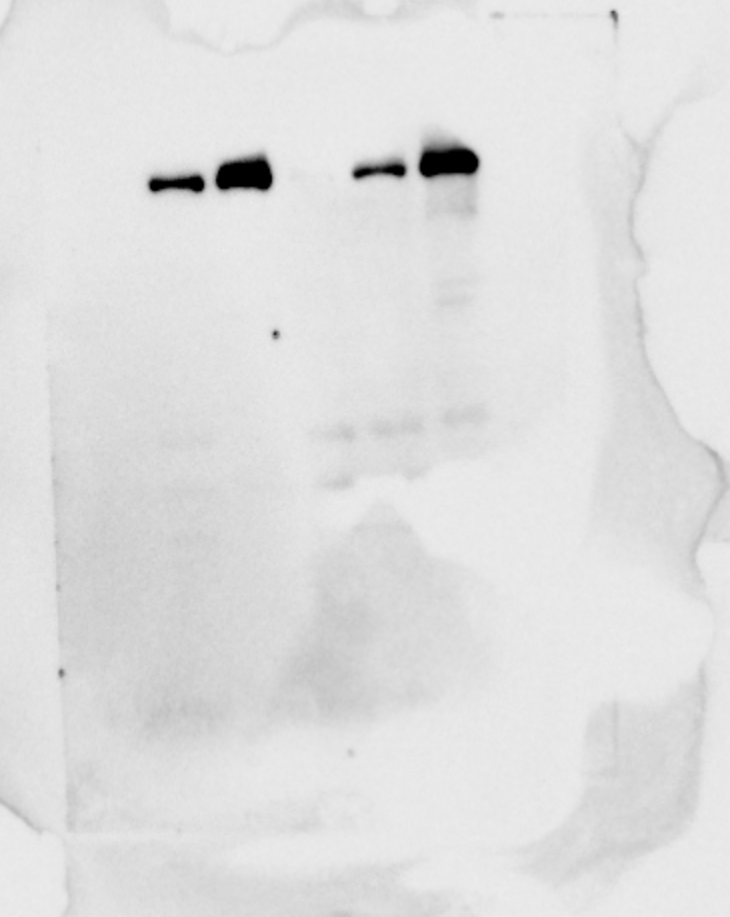

Supplement: Source data 1. [file elife-73628-data1.zip › Figure/Source data to Figure2 sup-figure 1/sup fig 1f/sup-fig 5f-p-TBK1-raw.png]

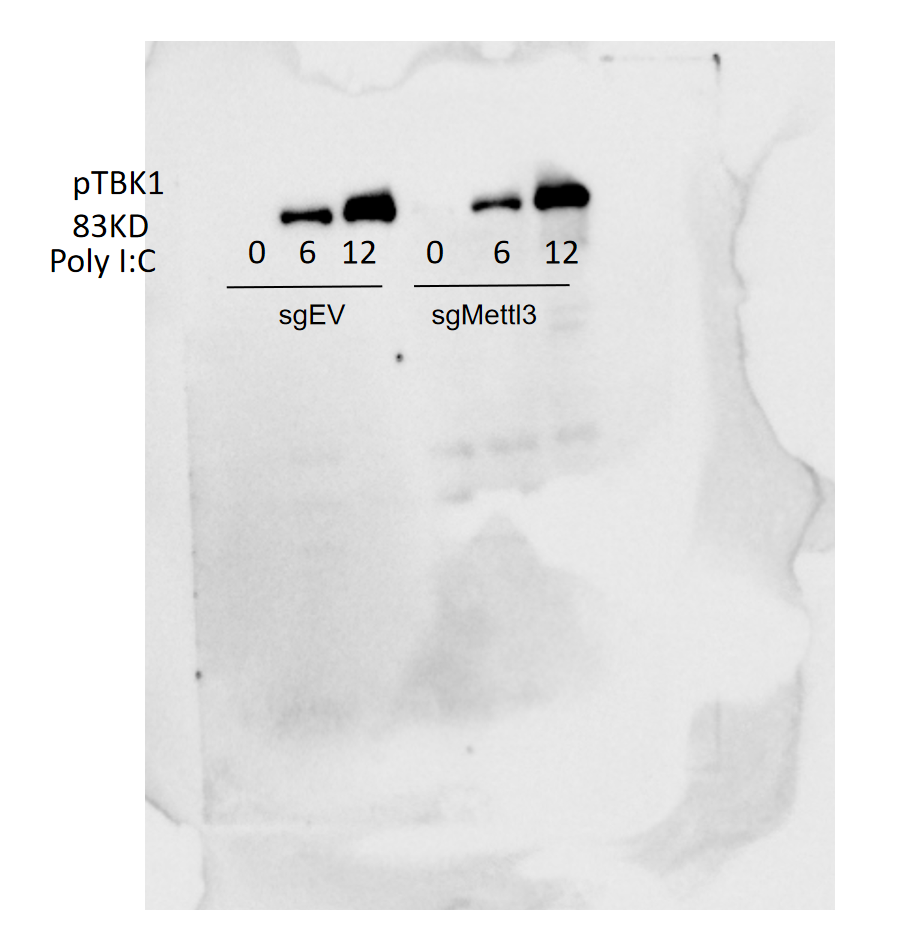

Supplement: Source data 1. [file elife-73628-data1.zip › Figure/Source data to Figure2 sup-figure 1/sup fig 1f/sup-fig 5f-p-TBK1.png]

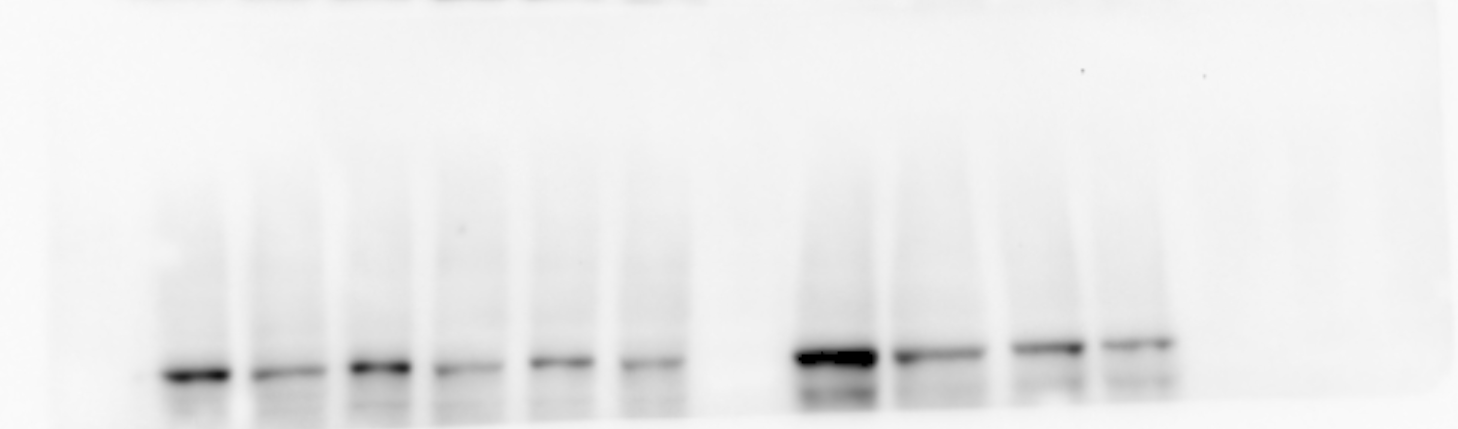

Supplement: Source data 1. [file elife-73628-data1.zip › Figure/Source data to Figure2 sup-figure 1/sup-fig 1d-Mettl3-raw.png]

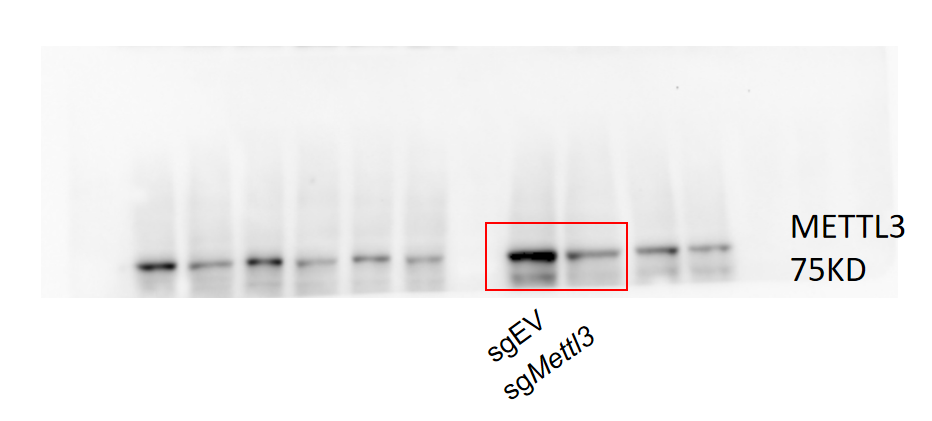

Supplement: Source data 1. [file elife-73628-data1.zip › Figure/Source data to Figure2 sup-figure 1/sup-fig 1d-Mettl3.png]

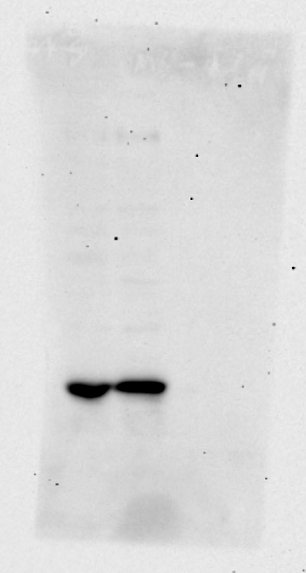

Supplement: Source data 1. [file elife-73628-data1.zip › Figure/Source data to Figure2 sup-figure 2/sup-figure 2b-ACTIN-raw.png]

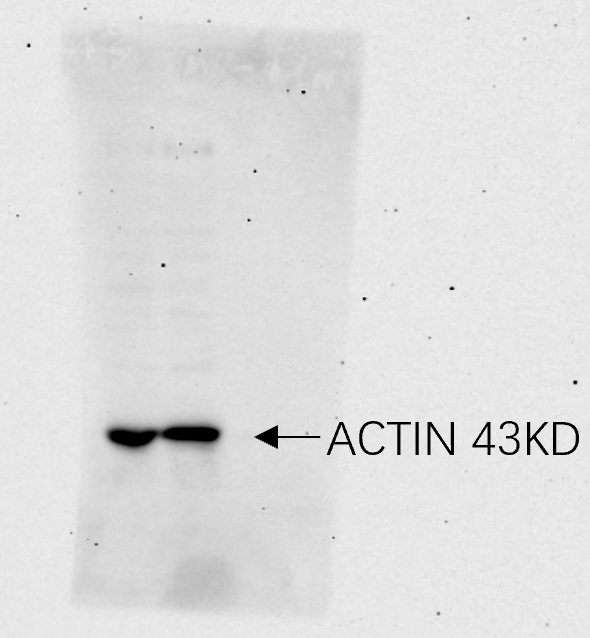

Supplement: Source data 1. [file elife-73628-data1.zip › Figure/Source data to Figure2 sup-figure 2/sup-figure 2b-ACTIN.png]

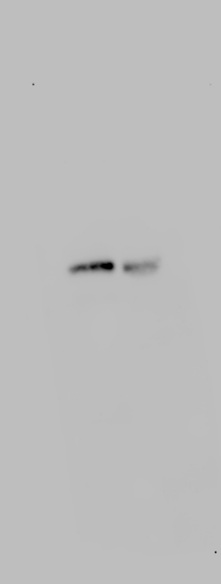

Supplement: Source data 1. [file elife-73628-data1.zip › Figure/Source data to Figure2 sup-figure 2/sup-figure 2b-Mettl3-raw.png]

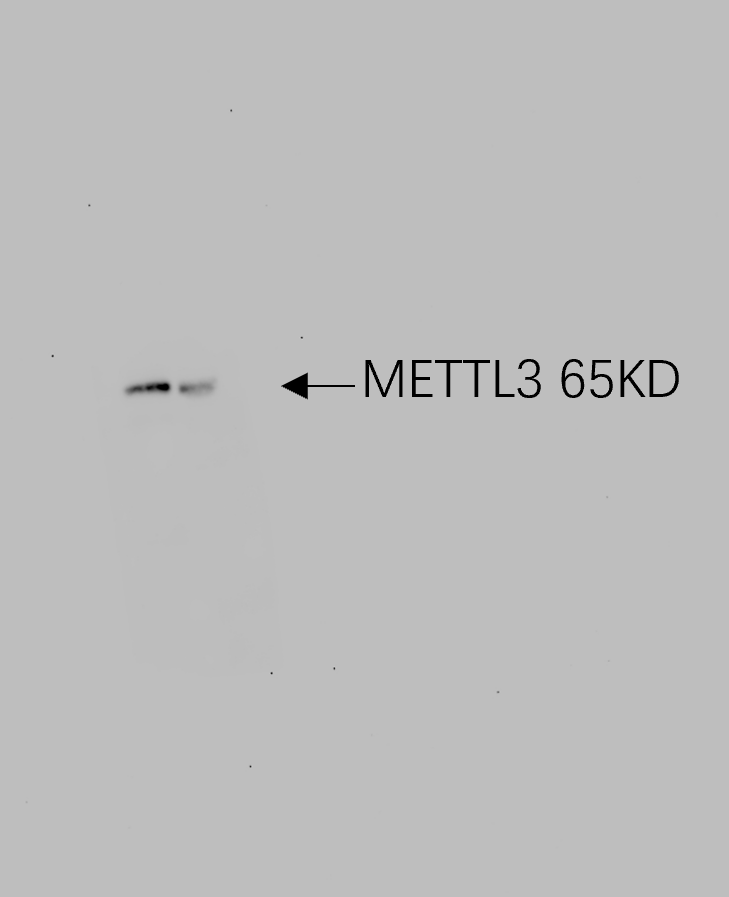

Supplement: Source data 1. [file elife-73628-data1.zip › Figure/Source data to Figure2 sup-figure 2/sup-figure 2b-Mettl3.png]

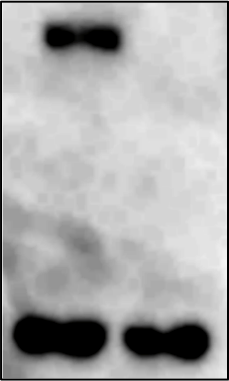

Supplement: Source data 1. [file elife-73628-data1.zip › Figure/Source data to Figure2 sup-figure 3/sup-figure 3b raw.png]

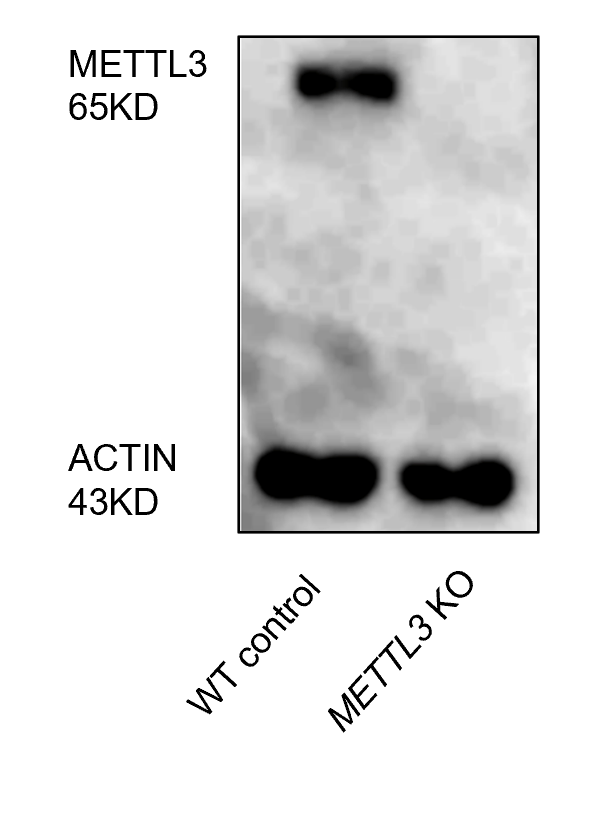

Supplement: Source data 1. [file elife-73628-data1.zip › Figure/Source data to Figure2 sup-figure 3/sup-figure 3b.png]

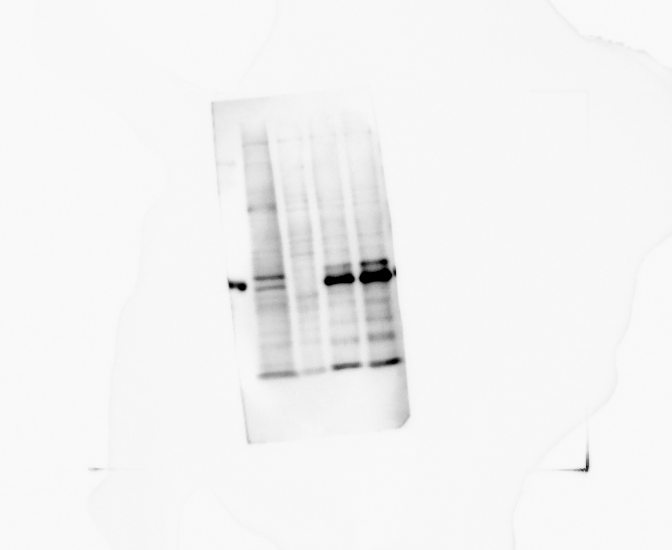

Supplement: Source data 1. [file elife-73628-data1.zip › Figure/Source data to Figure2 sup-figure 4/sup fig 4b-IRF7-raw.tif]

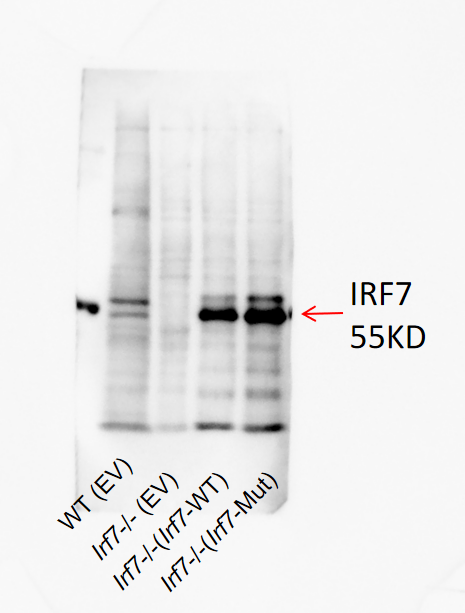

Supplement: Source data 1. [file elife-73628-data1.zip › Figure/Source data to Figure2 sup-figure 4/sup fig 4b-IRF7.png]

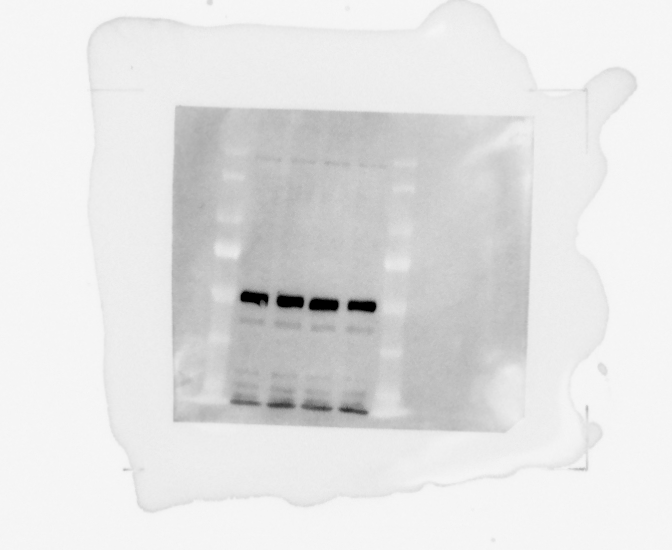

Supplement: Source data 1. [file elife-73628-data1.zip › Figure/Source data to Figure2 sup-figure 4/sup fig 4b-TUBULIN-raw.tif]

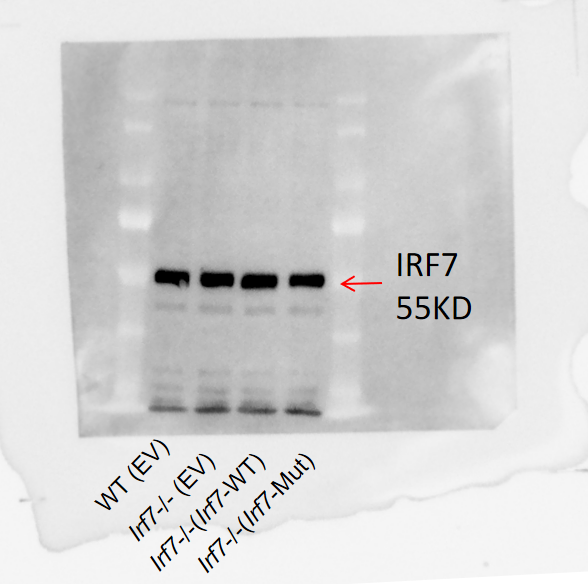

Supplement: Source data 1. [file elife-73628-data1.zip › Figure/Source data to Figure2 sup-figure 4/sup fig 4b-tubulin.png]
